# Improved Efficiency for Partial Oxidation of Methane by Controlled Copper Deposition on Surface‐Modified ZSM‐5

**DOI:** 10.1002/cctc.201500980

**Published:** 2015-12-04

**Authors:** Thomas Sheppard, Helen Daly, Alex Goguet, Jillian M. Thompson

**Affiliations:** ^1^ School of Chemistry and Chemical EngineeringQueen's UniversityStranmillis RoadBelfastBT9 5AGNorthern Ireland; ^2^ Institute of Catalysis Research and Technology (IKFT)Karlsruhe Institute of Technology (KIT)Kaiserstrasse 1276131KarlsruheGermany

**Keywords:** Cu-ZSM-5, methane, methanol, partial oxidation, silylation

## Abstract

The mono(μ‐oxo) dicopper cores present in the pores of Cu‐ZSM‐5 are active for the partial oxidation of methane to methanol. However, copper on the external surface reduces the ratio of active, selective sites to unselective sites. More efficient catalysts are obtained by controlling the copper deposition during synthesis. Herein, the external exchange sites of ZSM‐5 samples were passivated by bis(trimethylsilyl) trifluoroacetamide (BSTFA) followed by calcination, promoting selective deposition of intraporous copper during aqueous copper ion exchange. At an optimum level of 1–2 wt % SiO_2_, IR studies showed a 64 % relative reduction in external copper species and temperature‐programmed oxidation analysis showed an associated increase in the formation of methanol compared with unmodified Cu‐ZSM‐5 samples. It is, therefore, reported that the modified zeolites contained a significantly higher proportion of active, selective copper species than their unmodified counterparts with activity for partial methane oxidation to methanol.

## Introduction

Partial oxidation of methane to methanol represents a significant challenge in modern chemistry.^1^ Accomplishing this difficult reaction would facilitate the production of valuable chemical raw materials from methane abundant in natural reserves. In addition, conversion of natural gas into liquid form would simplify the issue of storage and transportation from remote or offshore sources.^2^ Although interest in this process has been high among the scientific community for many years, the partial oxidation of methane has proven difficult owing to the extreme stability of the methane molecule and the energy required to activate the C−H bond. Additionally, as the products of methane oxidation are inevitably more reactive than methane, total oxidation is a concern in any reaction system. Numerous examples of partial methane oxidation are available in the literature, but in many cases these require high temperatures, harsh solvents, or expensive reagents to facilitate the process. Catalytic oxidation to formaldehyde was demonstrated by Herman et al. over V_2_O_5_/SiO_2_, but with high temperature (600 °C) and low product selectivity towards CH_2_O (16.3 %) and CH_3_OH (2.5 %).^3^ Periana et al. used bipyrimidyl Pt^II^ complexes dissolved in concentrated H_2_SO_4_ to protect the product from further oxidation, obtaining methyl bisulfate with 81 % selectivity, yielding methanol and regenerating the catalyst by hydrolysis.^4^ However, the corrosive medium and homogeneous system remained problematic for product isolation. Later, Schüth et al. immobilised similar platinum catalysts on a polymer framework in a heterogeneous system allowing simple separation of products, but the corrosive acid medium was retained.^5^ Despite numerous advances, to date an efficient and viable method for direct conversion of methane to methanol remains elusive.

In the last decade, interest has shifted towards metal‐exchanged zeolites for the partial oxidation of methane to methanol, following the identification of a reactive copper species now characterised as the bent mono(μ‐oxo) dicopper core.^6^, ^7^, ^8^ The core was found to facilitate methane oxidation under mild conditions and can be stabilised in the framework of ZSM‐5 and other zeolites. The same copper species has been linked to the active site of the enzyme partial methane monooxygenase (pMMO), which offers an ideal mild reaction pathway for methane to methanol conversion in nature.^9^, ^10^ Besides Cu‐ZSM‐5, other zeolite frameworks including FER, BEA, MOR,^11^ and also artificial zeolites such as SSZ‐13 and SAPO‐34,^12^ are known to be active for partial methane oxidation following copper exchange. In particular, Cu‐FER and Cu‐MOR show variable activity for methane oxidation at different temperatures,^11^ leading to the assumption that other unidentified active copper cores may be present in various zeolite frameworks. Cu‐MOR is one of the most widely studied examples owing to its relatively high product yield and as yet undefined reaction mechanism,^13^, ^14^ although it is also known to contain the mono(μ‐oxo) dicopper core.^15^, ^16^ A growing body of research suggests a rich variety of species that may be active for partial methane oxidation, demonstrating the versatility of zeolites in supporting and stabilizing such species.^15^

In the present work, we focus on Cu‐ZSM‐5 as the earliest known example of a catalyst for partial methane oxidation to methanol with a copper‐exchanged zeolite. The original reaction method over Cu‐ZSM‐5 developed by Schoonheydt et al. is well documented.^11^ The zeolite is first activated in O_2_ at high temperature (350–650 °C); however, this can also be achieved at lower temperatures by using different oxidants such as NO^17^ or N_2_O.^11^ After activation, the reaction with methane proceeds under milder conditions at 150 °C to form methanol stoichiometrically with >98 % selectivity. This can be partially recovered ex situ by washing the catalyst with water or in situ by using steam, allowing an entirely gas‐phase process.^14^, ^18^ Following the interaction of methane with the activated catalyst, the product is thought to form initially as a methoxy intermediate, requiring a protic solvent in order to desorb.^8^, ^14^, ^15^ However, liquid‐phase extraction is known to be inefficient and some product remains trapped within the zeolite structure, possibly as a result of strong interactions with the numerous adsorption sites present and poor diffusion though the narrow channels of Cu‐ZSM‐5.^11^, ^19^ This behaviour has also been observed for Cu‐MOR,^12^ suggesting that the zeolite structure may play a role.^15^, ^20^ Alternatively, product extraction with steam at elevated temperatures (150–200 °C) has been shown to improve methanol yields in the case of Cu‐ZSM‐5,^17^ and allows complete desorption of products in the case of Cu‐MOR.^18^ It should be emphasised that partial methane oxidation over copper zeolite systems is stoichiometric,^11^ although batch‐type systems for recurring yields of methanol have been demonstrated.^17^, ^18^ Recently, Hutchings et al. detailed both batch and continuous‐flow liquid‐phase processes over bimetallic Fe/Cu‐ZSM‐5 (in which iron is the active species) by using H_2_O_2_ as the oxidant; however, the relatively expensive oxidizing agent and low conversion are typically problematic for partial methane oxidation.^21^, ^22^

Studies of Cu‐ZSM‐5 involving selective adsorption of IR probe molecules have identified at least two distinct copper environments; those on the external zeolite surface and those within the microporous framework.^19^ Only the latter have shown activity for low temperature, selective partial methane oxidation, indicating a relatively low proportion of active copper sites in the zeolite structure, with estimates at around 5 %.^11^ In the case of Cu‐ZSM‐5, it is thought that the numerous unselective or inactive copper sites contribute to inefficient product extraction and are potentially active for the total oxidation of methane to CO_2_.^7^, ^8^ Large volumes of inactive metal also complicate direct characterization by techniques such as X‐ray absorption spectroscopy (XAS), which measure the bulk metal species and thus have difficulty in distinguishing between suspected active sites and inactive metal sites.^23^, ^24^ The latter point is particularly relevant given that active copper sites across a range of zeolite types are still being discovered and characterised.^13^, ^14^, ^25^, ^26^, ^27^ Similarly, the precise activation mechanism of Cu‐ZSM‐5 with various oxidizing agents is still being debated.^15^, ^20^ The need for further detailed in situ or operando studies is therefore clear.

This work describes efforts to reduce the proportion of either the unselective or inactive copper within the structure of Cu‐ZSM‐5 through selective modification of the zeolite surface. Ichikawa et al. showed that interaction of a silylating agent with Brønsted acid zeolite exchange sites followed by high temperature treatment leads to passivation, forming Lewis acid SiO_*x*_ species and rendering the sites inactive for cation exchange.^28^ Passivation of zeolite exchange sites has already been applied for selective ion exchange on a number of metal–zeolite systems, including ZSM‐5. For example, Lercher et al. showed that the acidity of the external surface of ZSM‐5 could be decreased by modifying the external surface hydroxyl groups with bulky silylating agents.^29^ Alternatively, Iglesia et al. used the same technique to reduce the exchange of MoO_*x*_ species on the external surface of ZSM‐5, resulting in higher selectivity for the desired low‐order aromatics as a result of reactions occurring in the pores of the catalyst, as well as higher conversion owing to improved dispersion.^30^ Surface modification of various zeolites for selective catalysis on a variety of organic species is well established,^31^, ^32^, ^33^ but to the best of our knowledge has not previously been applied to the partial oxidation of methane to methanol.

Herein, selective functionalization of external zeolite exchange sites was performed with the bulky organic silylating agent bis(trimethylsilyl) trifluoroacetamide (BSTFA). The decrease in the total available exchange sites is intended to minimise formation of unselective copper nanoparticles on the external zeolite surface as well as promote exchange of copper within the zeolite pores during wet ion exchange, owing to an increased concentration gradient. The modified catalysts produced were structurally characterised and tested for activity towards partial methane oxidation. A number of unmodified catalysts were also prepared for comparison. The overall goal was to produce catalysts with an increased ratio of active and selective intraporous copper sites to unselective or inactive copper sites on the external zeolite surface, while retaining activity towards partial methane oxidation.

## Results and Discussion

### Diffuse reflectance infrared Fourier transform spectroscopy (DRIFTS) analysis of silylated zeolites

**Figure 1:**
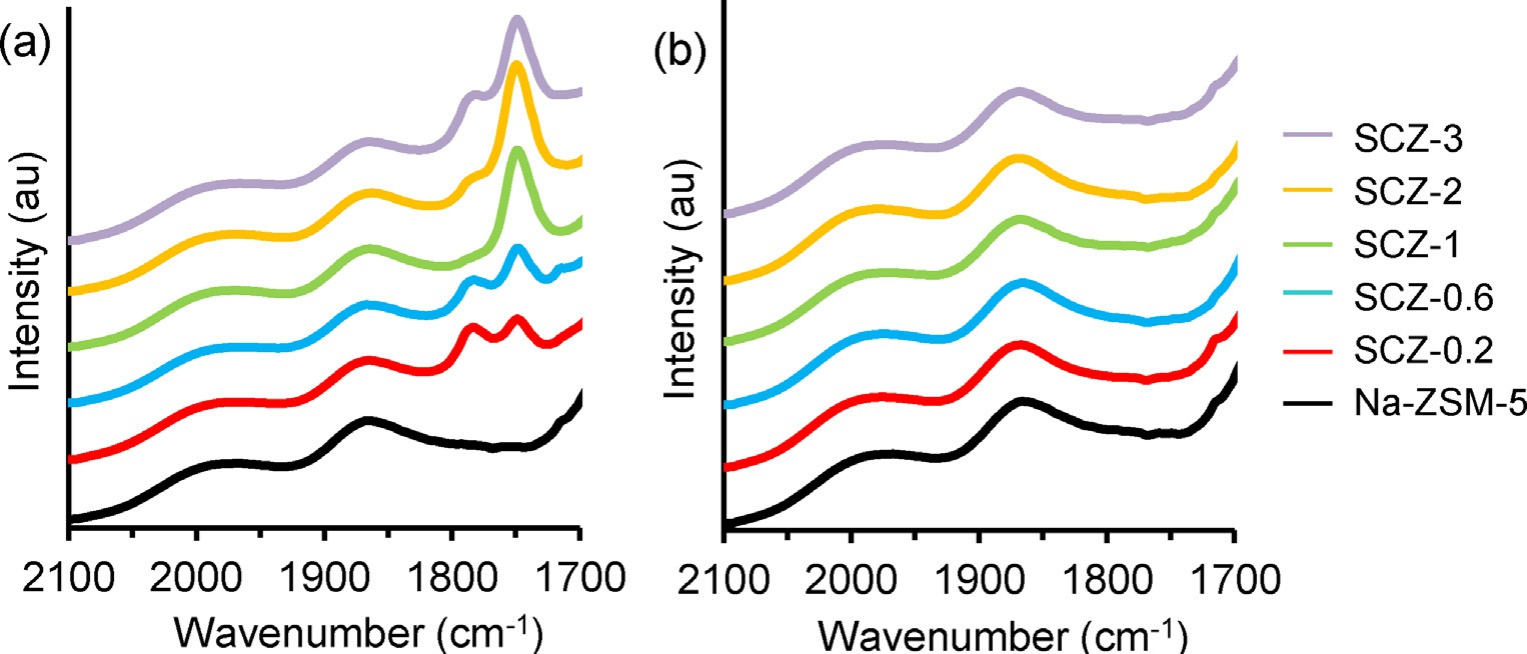
Zeolite overtones and carbonyl region (1700–2100 cm^−1^) of the DRIFTS spectra of BSTFA‐modified Na‐ZSM‐5 (a) after drying at 80 °C and (b) after calcination at 500 °C. Unmodified Na‐ZSM‐5 is shown for reference.

A range of modified Na‐ZSM‐5 samples were synthesised with SiO_2_ loadings ranging from 0.2 to 3 wt % (Table 1). The label ‘SCZ‐Y’ indicates a silylated Na‐ZSM‐5 zeolite with ‘Y’ loading of SiO_2_. Figure 1 (and Figure S4.1 in the Supporting Information) show the DRIFTS spectra of the samples following silylation, drying at 80 °C and calcination at 500 °C, in comparison to unmodified Na‐ZSM‐5. On treatment with BSTFA and drying, the carbonyl stretching band at 1745 cm^−1^ appeared with increasing intensity as silylation was increased, indicating functionalization of zeolite surface sites with BSTFA.

**Table 1:** Synthesis and physical characteristics of Cu‐ZSM‐5 zeolites.

Zeolite^[a]^	[Cu solution]	Cu	BSTFA	SiO_2_
	[mol dm^−3^]	[wt %]	[mol]	[wt %]
CZ‐0.7	5.00×10^−4^	0.70	–	–
CZ‐1.52	1.00×10^−3^	1.52	–	–
CZ‐1.88	1.25×10^−3^	1.88	–	–
CZ‐2.12	1.50×10^−3^	2.12	–	–
CZ‐2.65	2.00×10^−3^	2.65	–	–
CZ‐2.68	2.50×10^−3^	2.68	–	–
CZ‐2.88	3.00×10^−3^	2.88	–	–
CZ‐2.95	5.00×10^−3^	2.95	–	–
CZ‐2.65	2.00×10^−3^	2.65	0	0
SCZ‐0.2	2.00×10^−3^	2.08	3.36×10^−5^	0.2
SCZ‐0.6	2.00×10^−3^	2.45	1.01×10^−4^	0.6
SCZ‐1	2.00×10^−3^	2.39	1.67×10^−4^	1
SCZ‐2	2.00×10^−3^	2.11	3.36×10^−4^	2
SCZ‐3	2.00×10^−3^	1.94	5.04×10^−4^	3

[a] Labels: CZ‐X, CZ=copper‐exchanged zeolite, X=Cu wt % (determined by ICP); SCZ‐Y, SCZ=silylated copper‐exchanged zeolite, Y=SiO_2_ wt % (theoretical).

Silylated Na‐ZSM‐5 samples were then calcined at 500 °C to remove the organic precursors and complete the surface modification process. Thermal decomposition of the organic species was noted by the removal of all carbonyl bands observed during prior functionalization with BSTFA (Figure 1 b). Copper ion exchange was then performed on the modified Na‐ZSM‐5 samples to form Cu‐ZSM‐5, followed by treatment in air at 500 °C to remove organic species (see Figure S4.2). Copper exchange was accompanied by a colour change in the catalyst from white to pale blue.

### Structural characterization

The Cu‐ZSM‐5 samples were analysed by inductively coupled plasma (ICP) techniques, which revealed a range of total copper loadings from 0.7 to 2.95 wt % Cu for the unmodified (CZ‐X) series (Table 1). The silylated catalysts were prepared with equal concentrations of copper solution to the unmodified CZ‐2.65 sample and a uniform reduction in Cu wt % was observed, which was generally more prominent at higher levels of silylation. The reduced copper loading with increased silylation could be expected from the decrease in the availability of zeolite exchange sites owing to surface passivation as well as decreased diffusion in the precursor.

**Figure 2:**
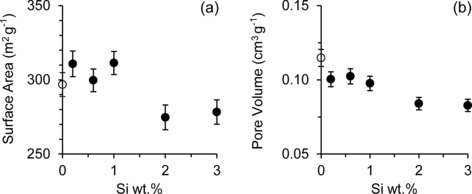
BET analysis of BSTFA‐modified Cu‐ZSM‐5 (0.2–3 wt % SiO_2_) and unmodified CZ‐2.65 (0 wt % SiO_2_, indicated by an unfilled circle): (a) total surface area; (b) cumulative pore volume.

Brunauer–Emmett–Teller (BET) analysis of the unmodified Cu‐ZSM‐5 samples showed approximate surface areas and pore volumes of 300–320 m^2^ g^−1^ and 0.109–0.115 cm^3^ g^−1^, respectively. This was within the expected range for microporous zeolites. For the modified Cu‐ZSM‐5 samples, a reduction in both surface area and pore volume was noted at higher (>1 wt % SiO_2_) levels of silylation (Figure 2). As no specific trend was identified for the unmodified samples (Section S3 in the Supporting Information), the trends observed are proposed to be a result of surface silylation. As BSTFA is larger than the pores of the zeolite (Figure S8.6), the decrease in surface area combined with lower Cu loading indicates that large amounts of newly formed SiO_2_ species on the zeolite surface reduce both the availability of the external exchange sites through passivation, and possibly intraporous exchange sites through steric blocking. However, the reduction in cumulative pore volume with increasing SiO_2_ wt % suggests increased exchange of copper within the zeolite channels. TEM performed on unmodified CZ‐2.12 revealed the presence of copper particles, which were visibly less numerous than for modified SCZ‐1 (Figure S3.1). Owing to the small channel size of ZSM‐5 (5.2–5.5 Å), visible particles of several nanometres were likely indicative of external surface species.^19^ Energy‐dispersive X‐ray spectroscopy (EDX) analysis revealed the presence of copper where none was physically visible, indicating high dispersion of metal nanoparticles for the silylated sample.

### FTIR analysis of copper distribution

As ICP results are only indicative of the total copper loading, the relative proportion of copper sites on the external surface and within the pores of Cu‐ZSM‐5 was estimated by using transmission IR. IR studies were performed with sequential adsorption of two probe molecules onto Cu‐ZSM‐5; firstly, pivalonitrile (PVN), which was considered too bulky to enter the zeolite channels and therefore adsorbs onto Cu on the external surface, followed by NO, which could access the remaining available copper sites in the channels. These probe molecules have previously been shown to be selectively indicative of external and intraporous copper species, respectively, with the amount of intraporous Cu proportional to the amount of methanol formed.^19^ Given that only copper sites in the zeolite channels were shown to be potentially active, the ratio of the integrated NO–Cu^2+^ to PVN–Cu^2+^ band intensities was used as an indicator of selective formation of intraporous copper species, with a maximised ratio being more favourable.

**Figure 3:**
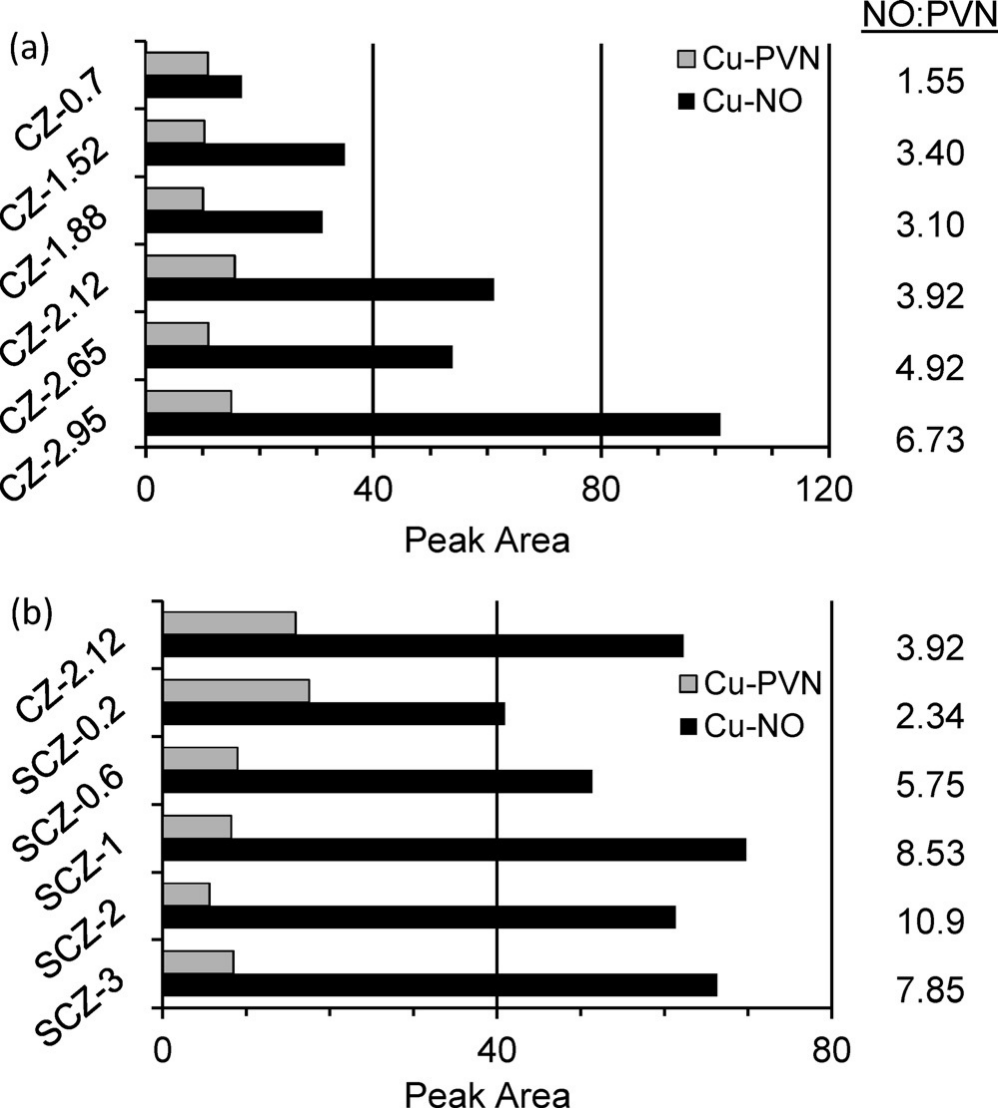
Integrated IR absorption peaks of PVN–Cu^2+^ (2280 cm^−1^) and NO–Cu^2+^ (1907 cm^−1^) following sequential adsorption of PVN then NO onto: (a) unmodified CZ series catalysts; (b) modified SCZ series catalysts (including CZ‐2.12 as 0 wt % SiO_2_ blank).

On exposure to PVN, a complex absorption feature was observed from 2210 to 2315 cm^−1^ (Figures S6.2 and S6.3). Contributions from PVN on the zeolite support were determined by following PVN adsorption on standard Na‐ZSM‐5. The PVN–Cu^2+^ absorption band was therefore assigned as that at 2280 cm^−1^ in accordance with Bitter et al.^19^ NO adsorption resulted in a second complex band at approximately 1900 cm^−1^ (Figure S6.4), which was isolated as above to identify the NO–Cu^2+^ band as that at 1907 cm^−1^. The key absorption bands at 2280 and 1907 cm^−1^ were deconvoluted and integrated to quantify the surface area of copper on the external and intraporous zeolite exchange sites, respectively. IR analysis results for the unmodified and modified catalyst series are shown in Figure 3 with the data summarised in Table 2.

**Table 2:** Catalytic activity and FTIR data for Cu‐ZSM‐5 zeolites for partial methane oxidation.

Zeolite	Cu	PVN–Cu	NO–Cu	NO/	UV/vis	Yield [μmol g^−1^]	
	[wt %]	[a.u.]	[a.u.]	PVN	[K–M]	GC	TPO
CZ‐0.7	0.70	11.1	17.2	1.55	0.14	0.00	1.71
CZ‐1.52	1.52	10.5	35.6	3.40	0.54	1.32	1.19
CZ‐1.88	1.88	10.2	31.6	3.10	0.95	1.97	–
CZ‐2.12	2.12	15.9	62.3	3.92	1.39	3.04	3.59
CZ‐2.65	2.65	11.1	54.8	4.92	2.20	2.56	10.4
CZ‐2.68	2.68	–	–	–	2.10	2.70	–
CZ‐2.88	2.88	–	–	–	2.67	2.75	–
CZ‐2.95	2.95	15.3	102.8	6.73	2.48	2.66	14.2
CZ‐2.12	2.12	15.9	62.3	3.92	1.39	3.04	3.59
SCZ‐0.2	2.08	17.5	40.9	2.34	0.52	1.13	2.73
SCZ‐0.6	2.45	8.94	51.4	5.75	1.12	1.64	6.32
SCZ‐1	2.39	8.18	69.8	8.53	0.69	1.01	13.0
SCZ‐2	2.11	5.61	61.4	10.9	0.70	1.42	11.1
SCZ‐3	1.94	8.45	66.3	7.85	0.53	1.51	6.56

Examination of the PVN–Cu^2+^ and NO–Cu^2+^ absorption peaks for the unmodified catalysts (CZ series) as a function of copper loading (Figure 3 a) indicates a similar peak area for PVN with increasing Cu loading, which implies similar volumes of Cu on the external zeolite surface, along with a significant increase in NO peak area, signifying an increase in the intraporous copper species. It has been shown that deposition of Cu onto the surface of the zeolites occurs rapidly during ion exchange (Figure S8.5) and so the relatively constant PVN signal can be attributed to rapid saturation of the external exchange sites, with slower diffusion of Cu into the pores. The increase in NO signal with higher Cu loading is likely due to the increased concentration gradients from the more concentrated Cu solutions, allowing more Cu to diffuse through to the interior zeolite channels. As the response factor of the probe molecules is unknown, it is unclear the extent to which the overall increase in the NO/PVN ratio observed is due to increased copper loading in the zeolite channels and how much is from a greater response factor for NO compared with PVN. Regardless, it is clear that there is an increase in the amount of Cu in the intraporous region of the zeolite with increasing Cu loading.

The modified catalyst series showed a simultaneous reduction in the PVN–Cu^2+^ signal and a general increase in the NO–Cu^2+^ signal with increasing silylation (Figure 3 b). As a constant copper concentration was used during preparation, these observations can therefore be attributed to the effects of silylation. Notably, a two‐fold increase in the NO/PVN ratio was observed for SCZ‐2 in comparison with the unmodified analogue CZ‐2.65. However, as the average copper loading of the modified catalysts was 2.19 wt % Cu, it is more appropriate to compare the structure and activity of the modified catalysts with that of CZ‐2.12, where a 2.8‐fold increase in NO/PVN was observed compared with SCZ‐2. It is apparent, therefore, that although the overall Cu loadings are similar, silylation has altered the distribution of Cu on the internal and external surfaces of the zeolite. An ideal silylation level of 1 to 2 wt % SiO_2_ is therefore suggested for this catalyst for maximizing the NO/PVN ratio to the implied optimum of active, selective copper sites compared with unselective copper sites.

### Activity testing for partial methane oxidation

The activity of the modified Cu‐ZSM‐5 samples towards partial methane oxidation was assessed. The catalysts were activated in oxygen and the presence of the UV/Vis band at 440 nm, representative of the bent mono(μ‐oxo) dicopper core, was determined.^11^ Following activation and reaction with methane, the strongly adsorbed reaction products were extracted with deionised water and analysed by GC. The results of the UV/Vis and GC analysis for both unmodified and modified catalyst series are shown in Table 2 along with the peak areas (arbitrary units) for adsorbed NO and PVN from the IR analysis. Yields are presented as μmol g^−1^ of zeolite used, unless stated otherwise.

**Figure 4:**
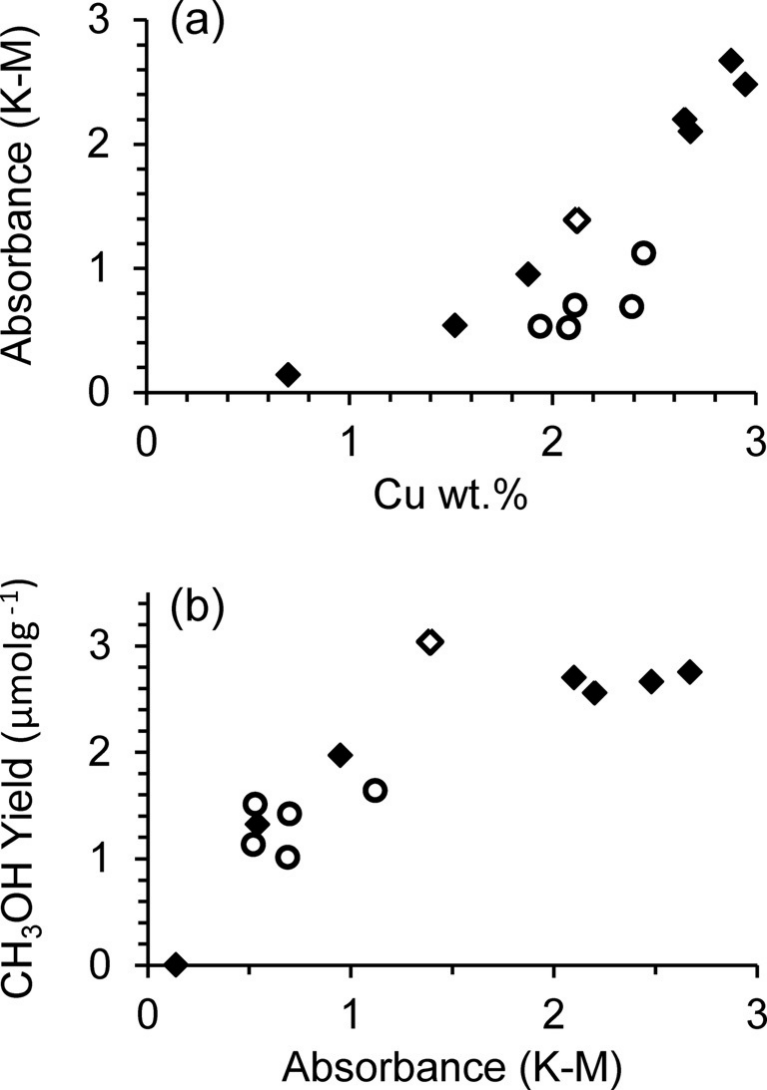
Comparison of modified and unmodified Cu‐ZSM‐5 samples: (a) UV/Vis intensity at 440 nm versus total copper loading (Cu wt %); (b) methanol yield after aqueous product extraction versus UV/Vis absorbance intensity at 440 nm. ♦=CZ series, ○=SCZ series, ◊=CZ‐2.12 (0 wt % SiO_2_ blank).

The 440 nm band was observed for all modified and unmodified catalysts tested following activation in O_2_ at 500 °C. For the unmodified CZ series, the intensity of the core band increased linearly with increasing copper loading (Figure 4 a), as observed by Schoonheydt et al.^11^ No methanol was observed below a minimum copper loading of 0.7 wt % Cu (0.14 K–M, Figure 4 b) and the methanol yield reached a maximum at 2.12 wt % Cu, (1.39 K–M, Figure 4 b) before decreasing slightly at higher copper loadings. Rather than limited formation of methanol, this plateau at high copper loadings has been attributed to the strong adsorption of product methanol to the catalyst and the high concentration of adsorption sites limiting aqueous methanol extraction.^8^, ^11^

For the modified SCZ series, a general decrease in UV/Vis signal intensity was observed compared with the unmodified CZ‐2.12 sample (Figure 4 a). Despite the small changes in Cu loading, a strong correlation with the methanol yield was not observed, rather a range of yields were observed from 1.0 to 1.6 μmol g^−1^. UV/Vis absorbance has previously been shown to be proportional to the amount of aqueous extracted product,^11^, ^19^ and the modified series also showed appropriate methanol yields in line with expectations from UV/Vis absorbance of the unmodified catalysts (Figure 4 b). This analysis shows that although the distribution of Cu changed after silylation, the modified catalysts were still active for partial methane oxidation. In addition, the ICP data indicate that the modified catalysts contained less Cu in total. Despite some samples giving a similar or greater NO–Cu^2+^ signal intensity compared with CZ‐2.12, implying greater amounts of copper in the pores, the UV/Vis data may suggest a lower than expected amount of core species was formed. However, the product extracted was in line with that expected from the amount of core species observed by UV/Vis.

**Figure 5:**
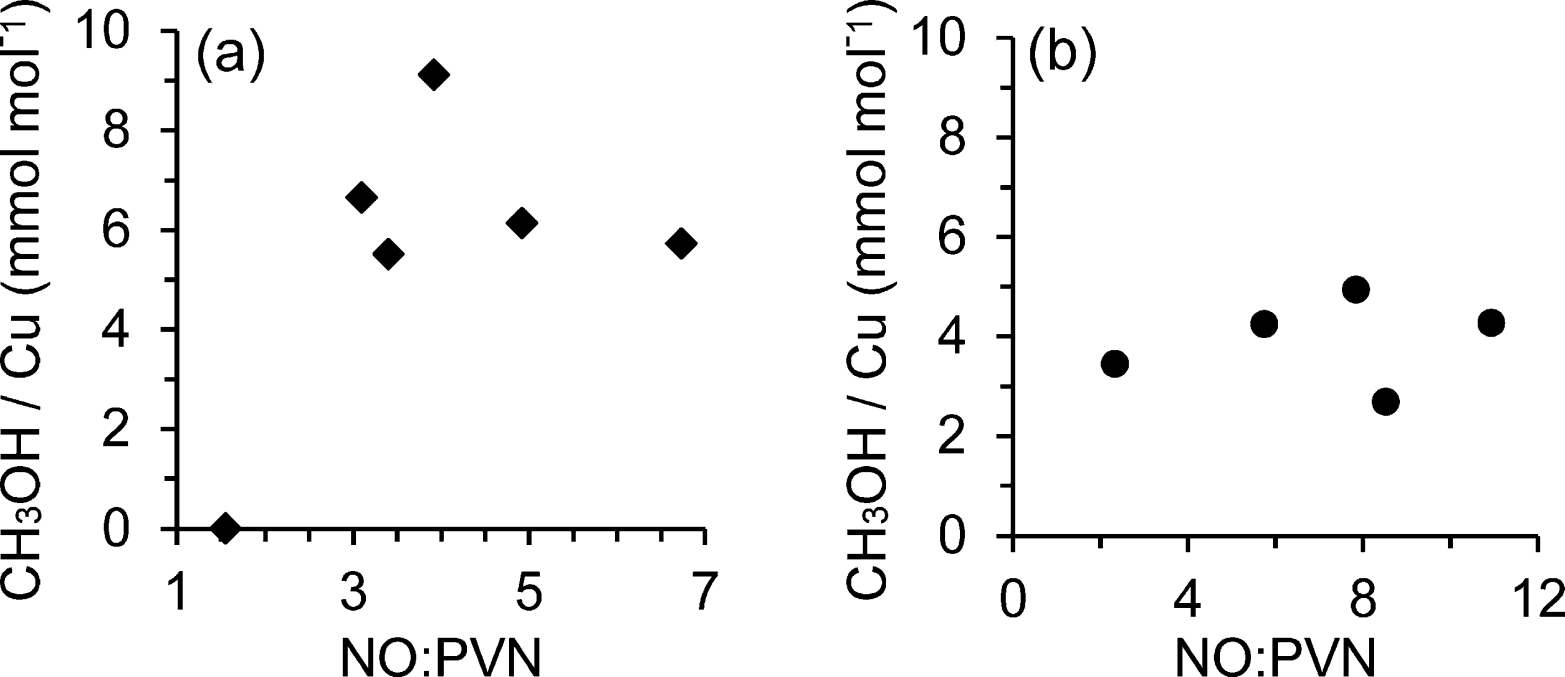
Methanol yield from aqueous extraction as a function of specific copper distribution on Cu‐ZSM‐5, characterised by integrated IR peaks following PVN and NO adsorption. Yields shown per moles of Cu for: (a) CZ series; (b) modified SCZ series.

Examining the aqueous product yield in relation to specific copper distribution (Figure S8.1) confirmed that the externally based copper sites characterised by PVN adsorption have no correlation with the methanol yield for either unmodified^19^ or modified zeolites. In contrast, intraporous copper sites characterised by NO adsorption have a clearly defined relationship with product yield for the unmodified series. Notably, the NO/PVN ratio for unmodified catalysts (Figure 5 a) reflects the relationship between the UV/Vis absorbance and yield shown previously (Figure 4 b). As the NO–Cu^2+^ peak increases in intensity and the NO/PVN ratio increases to above a critical value of about 4 (approximately 2.12 wt % Cu), the methanol yield reaches a plateau. Again, this is possibly due to hindered product desorption or the increased number of potential adsorption sites.^8^, ^11^ For the modified series, no trend was observed for either PVN or NO absorption, and in turn no trend between the NO/PVN ratio and product yield (Figure 5 b) was observed.

Care must be taken when comparing the performance of the modified and unmodified samples using UV/Vis and GC analysis. As mentioned previously, limitations to aqueous product desorption have been observed on ZSM‐5 at very high copper loadings based on temperature‐programmed oxidation (TPO) analysis,^11^ and studies involving product desorption by steam.^17^, ^18^ Here, the Cu loadings of the modified catalysts are mostly above the limit where there is a linear relationship range between CH_3_OH extracted and Cu loading, as shown by the plateau in Figure 4. It is widely accepted that at higher Cu loadings outside this range, aqueous extraction is limited and does not accurately represent the amount of product formed.^8^, ^12^, ^15^ In addition, formation of methanol in a similar process over zeolites other than ZSM‐5 shows varying response with UV/Vis analysis. Cu‐MOR gives a greatly reduced UV/Vis signal compared with the amount of methanol formed, whereas Cu‐BEA and Cu‐FAU show activity for low temperature methane oxidation but no significant UV/Vis peak at all.^11^, ^12^, ^18^

To test the validity of the trends observed with GC and UV/Vis analysis, TPO experiments were performed to evaluate the potential amount of adsorbates present on the catalyst surface following activation and reaction with methane. Combustion products were observed by mass spectrometry (MS) beginning at 270–280 °C for all catalysts. CO_2_ and CO were of particular interest in quantifying the adsorbed organic species. For the unmodified CZ‐series catalysts, the CO_2_ detected from blank TPO experiments (catalysts pre‐treated in Ar, see Section S7) was subtracted from the final CO_2_ yield. Given the high temperature pre‐treatment of all catalyst samples, it was assumed that any CO_2_ or CO observed from activated catalysts was, therefore, a result of strongly adsorbed methanol formed during reaction with methane.

**Figure 6:**
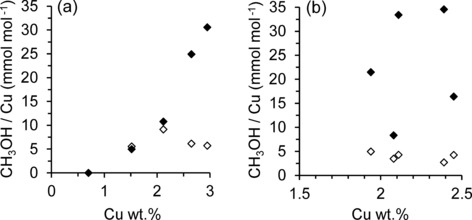
Activity data as a function of total copper loading. CH_3_OH yield after aqueous extraction (unfilled points) and CO_2_ evolved during TPO at 500 °C in O_2_ (solid points): (a) unmodified CZ series; (b) modified SCZ series.

Figure 6 shows the amount of methanol observed by GC following aqueous extraction compared with that observed by TPO‐MS as evolved CO_2_. For the unmodified series (Figure 6 a), below 2.12 wt % Cu the CH_3_OH and CO_2_ yields observed were very similar. However, at higher copper loadings a clear disparity was observed, whereby CO_2_ evolved was in excess of the aqueous desorbed products. For the modified series, a similar increase in product detected by TPO was observed compared with aqueous extraction (Figure 6 b). This confirms the limitation of aqueous product removal also for the silylated catalysts. At Cu loadings above approximately 1.9–2.1 wt % Cu, the volume of aqueous extracted product appears not to be an accurate representation of catalytic activity. Notably, CO or methanol desorption was not observed for any of the catalysts tested, indicating complete combustion of the adsorbed products in O_2_. CO_2_ and CH_3_OH yields are, therefore, considered as molar equivalents.

In Figure 6, product yields are expressed per moles of Cu present on the catalyst; this can effectively be considered as a turnover number (TON). However, it should be emphasised that the process shown here operates stoichiometrically; therefore, TON values are necessarily low and also the amount of copper does not represent the amount present in the active cores. However, comparing catalysts with similar copper loadings under this assumption permits useful comparison between the unmodified and modified series, as summarised in Table 3. The modified series, therefore, exhibited a maximum three‐fold increase in TON at an ideal silylation level of 1–2 wt % SiO_2_, and a two‐fold increase at 3 wt % SiO_2_. The former two catalysts outperformed even the most productive unmodified sample, CZ‐2.95. Considering the near identical copper loading observed between SCZ‐2 and CZ‐2.12, this increase in catalyst efficiency is particularly notable, indicating a significant increase in the amount of active catalytic sites present.

**Table 3:** Effect of silylation on TON and catalyst efficiency.

Zeolite	Cu	TPO yield^[a]^	TON^[b]^	Increase in
	[wt %]	[μmol g^−1^]	[mmol mol^−1^]	TON [%]^[c]^
CZ‐0.7	0.7	1.71	–	–
CZ‐1.52	1.52	1.19	4.97	–
CZ‐2.12	2.12	3.59	10.8	–
CZ‐2.65	2.65	10.4	24.9	–
CZ‐2.95	2.95	14.2	30.6	–
SCZ‐0.2	2.08	2.73	8.33	–
SCZ‐0.6	2.45	6.32	16.4	51.8
SCZ‐1	2.39	13.0	34.5	320
SCZ‐2	2.11	11.1	33.4	309
SCZ‐3	1.94	6.56	21.5	199

[a] Yield defined as μmol g^−1^ zeolite used. [b] TON defined as mmol CO_2_ observed per mol Cu present. [c] Compared with CZ‐2.12 as 0 wt % SiO_2_ blank.

**Figure 7:**
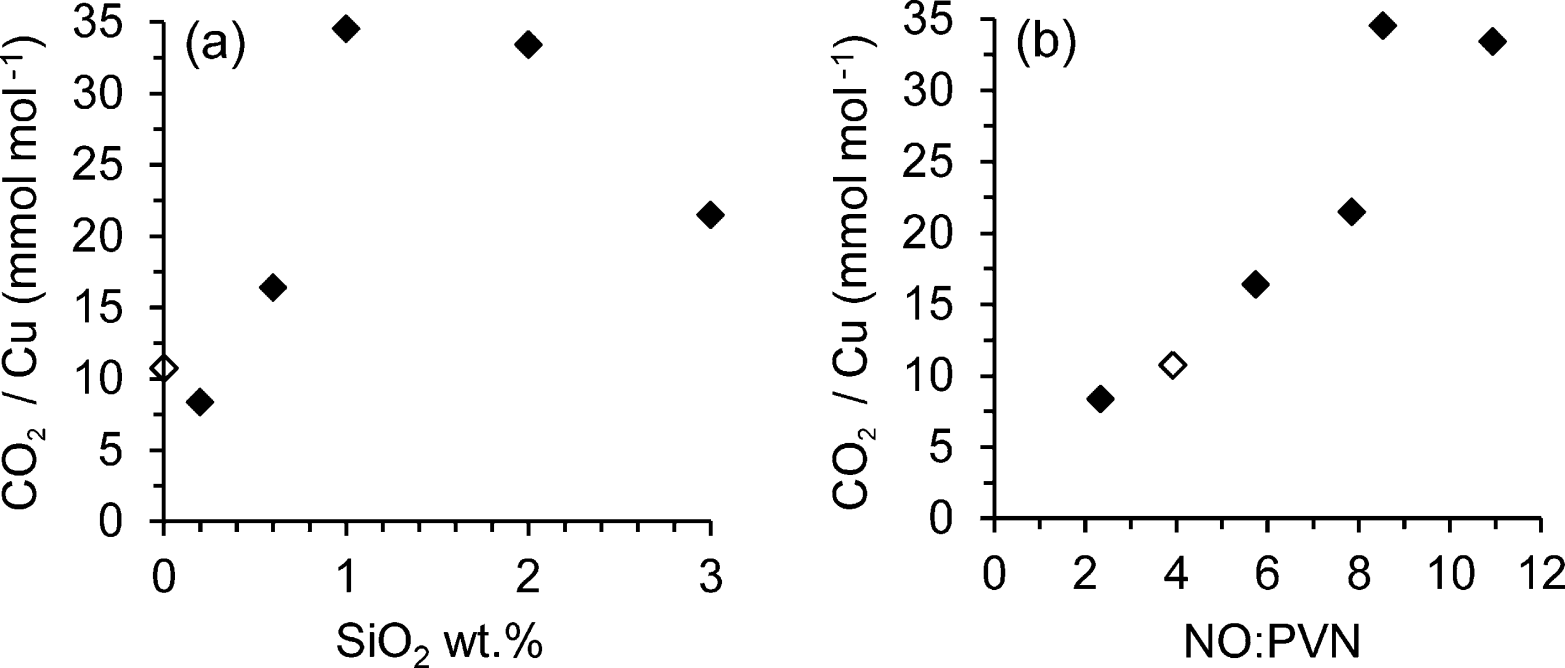
Activity data for modified SCZ series compared with CZ‐2.12 (0 wt % SiO_2_ blank, unfilled point). CO_2_ evolved during TPO at 500 °C in O_2_ in relation to: (a) % of silylation; (b) NO/PVN ratio as determined by integrated FTIR peaks.

It is also important to note that for the modified samples, no specific trend was observed between yield or TON and the total copper loading (Figure 6 b). However, by considering the level of silylation of the zeolite (Figure 7 a and Figure S8.3), it becomes clear that TON values are optimised at 1–2 wt % SiO_2_. Furthermore, this correlates well with the maximum value of NO/PVN observed through transmission FTIR analysis (Figure 7 b). As the volume of intraporous copper characterised by NO adsorption was found to correlate with the volume of product observed and TON (Figure S8.2), the activity of Cu‐ZSM‐5 for partial methane oxidation is therefore confirmed to be dependent only on the volume of copper in the zeolite channels. In agreement with the work of Bitter et al.,^19^ external copper sites were found to be inactive. It is apparent that the large increase in TON observed at 1–2 wt % SiO_2_ occurs as a direct result of silylation, which in turn offers an optimised amount of intraporous copper species that are potentially active.

In summary, for Cu‐ZSM‐5, the amount of aqueous extracted methanol does not accurately describe product formation above a certain Cu loading (2.12 wt % Cu for CZ‐series) and, therefore, the UV/Vis signal intensity does not correlate with yield above this value (Figure 4 b). However, for unmodified catalysts, the amount of CO_2_ evolved does correlate with the intensity of the NO and UV/Vis signal (Figures S8.2 and 8.4), indicative of active copper species. For the modified SCZ series catalysts, only the volume of CO_2_ evolved was found to correlate with the number of intraporous copper sites (Figure 7 b and Figure S8.2). As the CO_2_ evolved was in excess of the aqueous methanol yield, the CH_3_OH yield was not regarded as an accurate representation of product formation for any of the samples tested, any correlation with methanol yield was therefore not considered significant. Taking the CO_2_ evolved as being representative of the amount of product formed, the lack of correlation with UV/Vis data (Figure S8.4) shows this as not representative of the active species in the case of the modified catalysts. Examining the CO_2_ evolved together with FTIR analysis thereby provides a relationship between the intraporous Cu species and the amount of product formed, the effect of silylation was, therefore, assessed by using these parameters.

For the modified SCZ series, a clear influence was observed for the amount of CO_2_ evolved depending on silylation. At an ideal level of between 1–2 wt % SiO_2_ present on the zeolite surface, the CO_2_ yield reached a maximum, indicating a large amount of adsorbed methanol present within the zeolite pores, although product desorption in the aqueous phase remained significantly hindered, similar to the unmodified catalysts tested. The resulting modified zeolites, therefore, constitute more active catalysts than unmodified Cu‐ZSM‐5 based on the total copper loading present. The large increase in CO_2_ yield and TON at 1–2 wt % SiO_2_ is consistent with the structural characterization, which showed a greatly increased NO/PVN ratio during gas adsorption studies, marking the point at which the maximum intraporous copper sites and minimum external copper sites was observed. It should be noted that the trends observed began to reverse with extensive silylation of 3 wt % SiO_2_, which is possibly due to the maximum silylation level of the zeolite surface already being attained.^29^

This presents a strong case for applying silylation to selectively control copper deposition, showing that catalysts produced through surface passivation have a greater proportion of active, selective to non‐selective copper sites present than comparable unmodified samples. The increase in product yield can be directly linked to a change in specific copper distribution induced by silylation. The potential volume of product formed was easily determined by adsorption of gaseous probe molecules, a process that can be flexibly applied to other supports. A clear advantage of the silylation process described here lies in its applicability to other zeolite materials and framework types, along with the abundance of different silylating agents available. Application of this method to other framework types with partial methane oxidation activity, particularly those with greater pore diameters such as mordenite, may alleviate the problem of product desorption while resulting in a functional and more efficient catalyst. The increased proportion of active and selective copper present may also assist with further characterization of the active site.

## Conclusions

It has been shown that ZSM‐5 functionalised with the silylating agent BSTFA can facilitate passivation of specific zeolite exchange sites, allowing selective control over copper exchange during synthesis. A range of 1–2 wt % SiO_2_ was found to be ideal, effectively favouring deposition of copper within the zeolite channels at the expense of the external surface sites. With the observation that copper sites based on the external surface are inactive for low temperature partial methane oxidation, this indicates a significant increase in potentially active and selective copper within Cu‐ZSM‐5. Modified zeolites were found to be active for partial methane oxidation, showing product yields comparable with unmodified Cu‐ZSM‐5 samples based on UV/Vis analysis of the active copper species. TPO indicated that a large amount of methanol was formed for 1–2 wt % SiO_2_ catalysts, in direct proportion to the number of intraporous copper sites present. However, the product was not directly recoverable by aqueous extraction. Considering the CO_2_ yield in relation to the moles of Cu present, silylation of 1–3 wt % SiO_2_ provided a two‐ to three‐fold increase in TON over unmodified catalysts with comparable copper loading. Although the methane oxidation process demonstrated is stoichiometric, this indicates the increased efficiency of silylated catalysts for partial methane oxidation. The surface functionalization technique used is flexible and may be applied to a range of zeolite types and silylating species, advancing the prospect of developing a material consisting of a high proportion of active metal sites. This may help to facilitate direct characterization of the core species. In addition, application to zeolites with larger frameworks and pore diameters may contribute to developing more active and efficient catalysts for this important chemical process.

## Experimental Section

The synthetic parameters and characteristics of all zeolites produced are summarised in Table 1. The suppliers and purity of reagents and precursors are detailed in Section S1 in the Supporting Information. Cu‐ZSM‐5 samples were prepared by a standard wet ion exchange method.^11^ NH_4_‐ZSM‐5 (Si/Al=12, 1 g) was added to aqueous NaNO_3_ (1 g, 150 mL) and stirred for 24 h at room temperature (approximately 20 °C). Samples were filtered, washed and dried at 100 °C. Sodium exchange was performed three times to form Na‐ZSM‐5. Na‐ZSM‐5 (1 g) was added to solutions of Cu^II^ acetate monohydrate of various concentration in deionised water (250 mL) and the mixture stirred at room temperature for 24 h. Samples were filtered, washed and treated at 500 °C for 3 h to remove organic precursors before further use. Surface‐modified Cu‐ZSM‐5 samples were prepared by silylation of Na‐ZSM‐5 followed by copper ion exchange. Na‐ZSM‐5 (1 g) as prepared above was added to dry hexane (25 mL, 3 Å molecular sieve) and the mixture heated to 40 °C under a controlled nitrogen atmosphere. Varying concentrations of silylating agent BSTFA (3.73×10^−4^ to 3.73×10^−3^ mol L^−1^) in dry hexane were added and the mixture was heated at reflux for 1 h. Silylated samples were filtered, washed with dry hexane and dried at 80 °C for 1 h. Samples were then treated in air at 500 °C for 6 h to decompose the organosilane species and form silica‐modified Na‐ZSM‐5. Copper exchange was performed on the modified Na‐ZSM‐5 precursors as above, aiming to give a Cu loading similar to that of CZ‐2.65 (Table 1). Cu‐ZSM‐5 samples were treated again in air at 500 °C for 3 h before further use. Details of calculations for metal and BSTFA concentrations used are shown in Section S2 in the Supporting Information.

More details on the characterization techniques used and methods of deconvolution of peaks are shown in Sections S3–S6 in the Supporting Information. The Cu content of samples was determined by inductively coupled plasma optical emission spectrometry (ICP‐OES) by using a PE Optima spectrometer. BET surface areas and pore volumes of zeolite samples were determined by nitrogen adsorption at −196 °C by using a Micrometrics Tristar II instrument. TEM was performed by using an FEI Tecnai F20 electron microscope operated at 200 kV, following adherence of the samples to a copper microgrid. DRIFTS was performed at various stages during the synthesis to monitor the progress of silylation: (a) fresh Na‐ZSM‐5 before modification by silylation, (b) modified Na‐ZSM‐5 after drying, (c) modified Na‐ZSM‐5 after calcination, (d) modified Cu‐ZSM‐5 after copper exchange. Spectra were recorded at ambient temperature by using a Bruker Tensor 27 IR spectrometer at a resolution of 4 cm^−1^. Each spectrum was the result of 128 scans, with dry KBr used as the background. Spectra were baseline corrected and normalised to the zeolite overtones (1950–2050 cm^−1^). UV/Vis spectroscopy was used to monitor the presence of the active copper species in Cu‐ZSM‐5 during activation and reaction with methane.^11^ Pelletized samples (0.7 g, 250–500 μm pellets) were placed in a quartz plug flow reactor and activated in oxygen at 500 °C overnight. Spectra were recorded by a diffuse reflectance fibre optic probe (Hellma 668.006‐UVS) with a PerkinElmer Lambda 650S spectrometer, with the parent zeolite, NH_4_‐ZSM‐5, used as the background. Transmission FTIR was used in conjunction with the adsorption of probe molecules to monitor the distribution of copper on the samples.^19^ Cu‐ZSM‐5 samples (20 mg) were pressed into self‐supporting discs, placed in a Specac gas exchange cell and outgassed under vacuum at 150 °C for 2 h. The sample discs were then exposed to PVN vapour (30 min, ∼8 mbar) and subsequently NO (overnight, 25 mL min^−1^, 1 % in Ar) at 40 °C. Blank experiments were also performed by adsorption of PVN then NO to the parent zeolite Na‐ZSM‐5. During probe adsorption, spectra were recorded in situ in transmission mode on a Bruker Tensor 27 IR spectrometer at a resolution of 4 cm^−1^. Each spectrum was the result of 128 scans, with dry KBr used as the background. Spectra were baseline corrected and normalised to the zeolite overtones (1950–2050 cm^−1^). A deconvolution process was applied to isolate the absorption bands of interest (see Section S6 in the Supporting Information).

Standard and modified Cu‐ZSM‐5 samples were tested for activity towards partial methane oxidation (Section S7 in the Supporting Information). Cu‐ZSM‐5 samples (0.7 g, 250–500 μm pellets) were placed in a quartz plug flow reactor and activated overnight in oxygen (50 mL min^−1^, 500 °C). Samples were cooled to room temperature, flushed with argon and then exposed to methane (50 mL min^−1^, 1 % in Ar) at 150 °C for 1 h (10 °C min^−1^). The products remained adsorbed to the catalyst surface. Two different analytical methods were used on spent samples following activation and reaction with methane: (i) reaction products were directly extracted in aqueous solution and analysed by GC, (ii) TPO was performed and combustion products monitored by MS. By the GC method, spent samples were removed from the reactor and stirred vigorously in deionised water (1.5 mL) for 24 h. The suspension was centrifuged and the supernatant liquid passed through a syringe filter (0.45 μm, nylon membrane). The product composition was determined by using a PerkinElmer Clarus 500 GC equipped with a FID and a Supelco Carbowax Amine column (30 m×530 μm ID). By the TPO method, spent samples were directly heated from 20 to 500 °C (10 °C min^−1^) in oxygen (40 mL min^−1^) with krypton (10 mL min^−1^) as an internal standard and combustion/desorption products CO (*m*/*z*=28), CH_3_O^+^ (*m*/*z*=31) and CO_2_ (*m*/*z*=44) were monitored by using a calibrated Hiden Analytical MS HPR20.

## Supporting information

As a service to our authors and readers, this journal provides supporting information supplied by the authors. Such materials are peer reviewed and may be re‐organized for online delivery, but are not copy‐edited or typeset. Technical support issues arising from supporting information (other than missing files) should be addressed to the authors.

Supplementary
