# Supplementary material for: Improved Efficiency for Partial Oxidation of Methane by Controlled Copper Deposition on Surface‐Modified ZSM‐5
Source: ChemCatChem. 2015 Dec 4;8(3):562–70. doi: 10.1002/cctc.201500980 (PMC4755137; doi:10.1002/cctc.201500980)
Supplement: Supplementary file 1 — Supplementary [file CCTC-8-562-s001.pdf]

Heterogeneous & Homogeneous & Bio- & Nano-
CHEMCATCHEM
CATALYSIS

Supporting Information

Improved Efficiency for Partial Oxidation of Methane by Controlled Copper Deposition on Surface-Modified ZSM-5

Thomas Sheppard, Helen Daly, Alex Goguet, and Jillian M. Thompson*[a]

cctc_201500980_sm_miscellaneous_information.pdf

S1 - Materials

The ammonium form of ZSM-5 (NH_4-ZSM-5, MFI framework, Si/Al = 12) was supplied courtesy of Süd Chemie and was used as a precursor for synthesis of H-, Na- and Cu-ZSM-5 according to the methods described below. Molecular sieves (3 Å, Alfa Aesar) were rinsed with H_2O and treated in air at 500 °C before initial use.

The following chemicals used are listed with their purity, source and nomenclature: bis(trimethylsilyl)trifluoroacetamide (BSTFA , 98.7%, Supelco Analytical), copper(II) acetate monohydrate ($Cu(CH_3COO)_2$, ≥99.0%, Sigma-Aldrich), deionised H_2O (18 MΩ-cm , prepared on-site), hexane (C_6H_{14} , ≥99.0%, Fluka Analytical), methanol (CH_3OH , 99.99%, Fisher Scientific), pivalonitrile / trimethylacetonitrile (PVN , 98%, Acros Organics), sodium nitrate ($NaNO_3$, ≥99.5%, BDH).

All gases were obtained from BOC with the following composition and purity: argon (Ar, >99.99%), carbon dioxide (CO_2, 100%), helium (He, 99.9%), krypton (Kr, 5% in Ar), methane (CH_4, 1% in Ar), nitric oxide (NO, 1% in Ar), nitrogen (N_2, 99.9%), oxygen (O_2, 99.5%).

S2 - Catalyst Preparation

Cu-ZSM-5 samples were prepared by wet impregnation of ZSM-5 with copper solution and are given the nomenclature CZ-X, where 'CZ' indicates a copper-exchanged zeolite and 'X' denotes the total copper loading (Cu wt.%). NH_4-ZSM-5 (1 g) was added to a solution of $NaNO_3$ (1 g) in deionized H_2O (150 ml) and the mixture stirred at room temperature for 24 hours. The zeolite was recovered by vacuum filtration, washed with deionized water and dried at 100 °C. The sodium exchange procedure was repeated 3 times to form Na-ZSM-5. Na-ZSM-5 (1g) was then added to a solution of Cu(II) acetate monohydrate in deionized water (250 ml) and the mixture stirred at room temperature for 24 hours. The concentration of copper solution used was determined by target theoretical values of Cu wt.% and Cu/Al ratio (Figure S2.1). The zeolite was recovered by vacuum filtration, washed with deionized water and dried at 100 °C. All standard Cu-ZSM-5 samples were treated at 500 °C for 2 hours in a muffle furnace to remove organic precursors before further use. The preparation characteristics for standard Cu-ZSM-5 samples are summarized in Table S2.1.

Figure S2.1 - Theoretical Determination of Copper Loading for Cu-ZSM-5 Synthesis

ZSM-5 has the following structural formula:

$[(M)_x(H_2O)_{16}][Al_xSi_{96-x}O_{192}]$ where 'M' is the (+1) charge compensating cation

$[(M)_{0.5x}(H_2O)_{16}][Al_xSi_{96-x}O_{192}]$ where 'M' is the (+2) charge compensating cation

For Cu-ZSM-5 prepared directly from Na-ZSM-5, assuming non-saturation with Cu^{2+}:

$[(Na)_y(Cu)_z(H_2O)_{16}][Al_xSi_{96-x}O_{192}]$ where x = 2z + y

$[(Na)_{3.46}(Cu)_{1.97}(H_2O)_{16}][Al_{7.4}Si_{88.6}O_{192}]$ where Si/Al = 12

eg. 2 wt.% Cu^{2+} = 1.97 mol Cu^{2+} in 1 mol zeolite

= 3.15×10^{-4} mol Cu^{2+} in 1 g zeolite

Table S2.1 - Preparation of Standard Cu-ZSM-5

Zeolite	Cu^{2+} Acetate				Theoretical		Experimental	
	(g)	(mmol)	(ml)	(mmoldm^{-3})	wt.% Cu	Cu/Al	wt.% Cu	Cu/Al
CZ-0.7	0.025	0.125	250	0.50	0.8	0.105	0.7	0.106
CZ-1.52	0.050	0.250	250	1.00	1.59	0.211	1.52	0.233
CZ-1.88	0.062	0.313	250	1.25	1.99	0.264	1.88	0.280
CZ-2.12	0.075	0.375	250	1.50	2.38	0.316	2.12	0.382
CZ-2.65	0.100	0.500	250	2.00	3.17	0.423	2.65	0.382
CZ-2.68	0.125	0.625	250	2.50	3.74	0.5	2.68	0.379
CZ-2.88	0.150	0.750	250	3.00	3.74	0.5	2.88	0.397
CZ-2.95	0.250	1.250	250	5.00	3.74	0.5	2.95	0.377

Surface modified Cu-ZSM-5 samples were prepared by silylation of Na-ZSM-5 followed by wet impregnation with copper solution and are given the nomenclature SCZ-Y, where 'SCZ' indicates a silylated copper-exchanged zeolite and 'Y' denotes the loading of silylating agent (SiO_2 wt.%). Na-ZSM-5 (1 g) prepared as above was placed in a round-bottom flask under a nitrogen atmosphere. Dry hexane (25 ml, 3 Å molecular sieve) was added via septum and the mixture heated to 40 °C. The silylating agent BSTFA was added via septum and the

mixture refluxed for 1 hour. The concentration of BSTFA used was determined by target theoretical values of SiO_2 wt.% (Figure S2.2). The modified zeolite was recovered by vacuum filtration, washed with dry hexane and treated in air at 500 °C for 6 hours, to decompose the organosilane species present and form silica-modified Na-ZSM-5. Copper exchange was performed on the modified sodium-zeolite precursors as discussed previously. Following silylation and copper exchange, modified Cu-ZSM-5 samples were treated again in air at 500 °C for 3 hours before further use. The preparation characteristics for standard Cu-ZSM-5 samples are summarized in Table S2.2.

Silylation was considered in terms of wt.% SiO_2 according to Lercher *et al.*[29] For example, to obtain 4 wt.% SiO_2 loading with the silylating agent BSTFA on 1 g of zeolite:

$$1\ g\ zeolite, 4\ wt.\% = 0.04\ g\ SiO_2$$
$$n = \frac{m}{M} \quad \therefore \quad \frac{0.04}{60} = 0.000672\ mol\ SiO_2$$
$$6.72 * 10^{-4}\ mol\ SiO_2 \ \equiv \ 6.72 * 10^{-4}\ mol\ BSTFA$$
$$m = n * M \quad \therefore \ 0.000672 * 257.4 = 0.173\ g\ BSTFA$$
$$V = \frac{m}{D} \quad \therefore \quad \frac{0.173}{0.960} = 0.18\ ml\ BSTFA$$

Therefore 0.18 ml of undiluted BSTFA should be added to a mixture of zeolite (1 g) and hexane (25 ml), resulting in a silylation equivalent to 4 wt.% SiO_2.

Table S2.2 - Preparation of Surface Modified Cu-ZSM-5

Zeolite	Silylating Agent BSTFA				Cu^{2+} Acetate		Experimental	
	(ml)	Dilution Factor[a]	Sample Size[b] (ml)	Theoretical wt.% SiO_2	(ml)	(mmoldm^{-3})	wt.% Cu	Cu/Al
SCZ-0.2	0.09	1:10	0.090	0.2	250	2.00	2.08	0.275
SCZ-0.6	0.09	-	0.027	0.6	250	2.00	2.45	0.286
SCZ-1	0.09	1:1	0.090	1.0	250	2.00	2.39	0.280
SCZ-2	0.09	-	0.090	2.0	250	2.00	2.11	0.298
SCZ-3	0.09	-	0.135	3.0	250	2.00	1.94	0.260

[a]Indicates dilution of pure BSTFA with hexane, eg. '1:10' is equivalent to 0.09 ml BSTFA in 0.81 ml hexane. [b]Indicates the actual volume of BSTFA (pure or in hexane) in the reaction mixture.

S3 - Catalyst Characterization by ICP, BET and TEM

Elemental analysis was performed by fusing Cu-ZSM-5 samples (20 mg) with sodium peroxide (0.5 g) in a zirconium crucible at 500 °C. The molten samples were quenched with water and 2M HCl added to form a resultant solution of 20 ml. The solution was analysed by ICP-OES using a PE Optima spectrometer. The Si/Al ratio of the parent zeolite was confirmed as 12. Cu content and Cu/Al ratio of the standard and modified catalysts produced are shown in Table S2.1 and S2.2 respectively.

BET was used to determine surface area and pore volumes through N_2 adsorption using a Micrometrics Tristar II instrument. Samples were first heated at 100 °C for 1 hour followed by 350 °C for 2 hours under a stream of nitrogen to purge the zeolite surface of volatiles. BET analysis of the standard Cu-ZSM-5 catalysts showed approximately uniform surface area of 300 to 320 ± 5 m^2/g, a small reduction from the value of 380 m^2/g measured for the precursor NH_4-ZSM-5. This was attributed to the introduction of copper to the zeolite.[19] BET analysis of the silylated Cu-ZSM-5 samples is discussed further in the main paper.

TEM was performed using an FEI Tecnai F20 electron microscope equipped with a field emission gun (200 kV) and EDX accessory. Cu-ZSM-5 samples were suspended in 95% ethanol solution and adhered to a copper microgrid before imaging from 9.9-880 kx magnification. TEM images of an unmodified catalyst (CZ-2.12) and a catalyst with moderate silylation (SCZ-1) were examined. Dark spots were clearly visible on the surface of CZ-2.12 (Figure S3.1a), which revealed a highly ordered structure at high magnification (Figure S3.1b) indicative of copper particles. A range of particles could be easily observed with maximum diameter of approximately 10 nm which, given the diameter of the ZSM-5 micropores (5.2-5.5 Å), are likely confined to the external surface.[19] As SCZ-1 exhibited notable differences in pore volume and copper loading compared to the unmodified sample, attributed to passivation, it was thought that TEM analysis would reveal differences in copper distribution. Accordingly the characteristic dark spots indicative of copper were more sparse on SCZ-1 and were generally only observed at higher magnification (Figure S3.1d). Further analysis by EDX at several points on the sample revealed the presence of copper where none was physically visible, suggesting highly dispersed copper across the zeolite surface with fewer visible particles attributed to the external surface species, concurrent with surface passivation. The combination of physical characterization methods used therefore suggests distinct changes particularly in the zeolite surface structure as a result of silylation. This was investigated further through spectroscopic analysis.

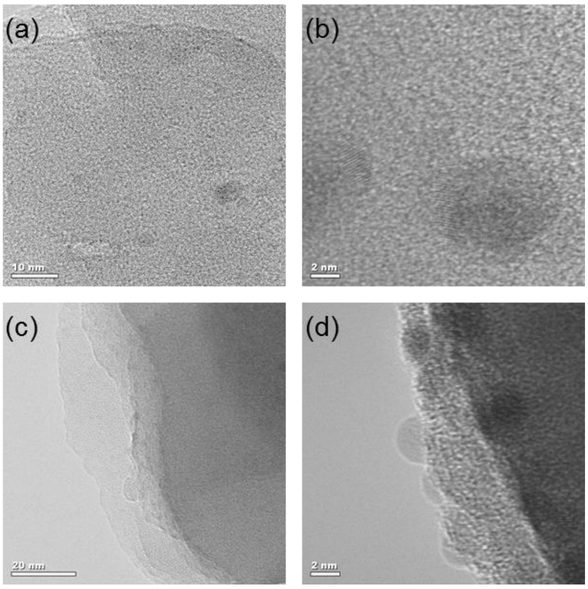

Figure S3.1 - TEM images of unmodified CZ-2.12 magnified at: (a) 285k; (b) 880k; and modified SCZ-1 magnified at: (c) 195k; (d) 880k.

S4 - DRIFTS Analysis of Silylated Catalysts

Diffuse Reflectance IR Spectroscopy (DRIFTS) was performed during synthesis to monitor the progress of silylation, by observing the presence of organosilane features and changes in zeolite silanol bands. Specifically, spectra were recorded for:

- fresh Na-ZSM-5 before silylation
- modified Na-ZSM-5 after drying
- modified Na-ZSM-5 after calcination
- modified Cu-ZSM-5 after copper exchange.

Powder samples were analyzed *ex-situ* at ambient temperature using a Bruker Tensor 27 IR spectrometer. Spectra were obtained from 400 to 4000 cm^{-1} with a resolution of 4 cm^{-1}. Each spectra was the result of 128 scans, with dry KBr powder used as background. Data processing was performed using the OPUS software package. All spectra were manually baseline corrected and normalized to the zeolite overtones at 1950-2050 cm^{-1}. Qualitative analysis of the resultant spectra was then performed to identify functional groups of interest and assess any changes in sample surface structure and bonding during and after silylation treatment.

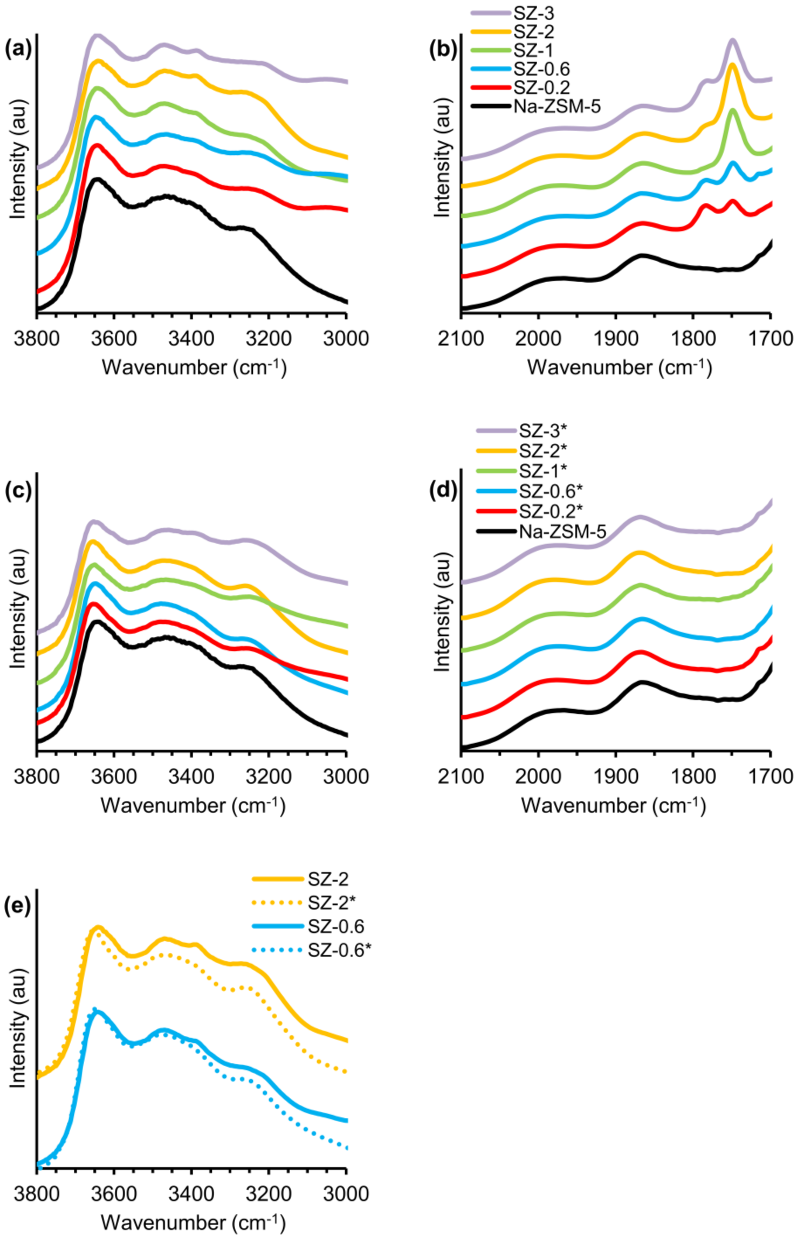

Figure S4.1 - DRIFTS spectra of BSTFA-modified Na-ZSM-5 after drying at 80 °C: (a) silanol and amide region; (b) zeolite overtones and carbonyl region. After calcination at 500 °C (indicated by *) (c) silanol and amide region; (d) zeolite overtones and carbonyl region. (e) A comparison of selected catalysts before (solid line) and after (dotted line) calcination at 500 °C. A spectrum of unmodified Na-ZSM-5 is shown for reference.

Figure S4.1 shows DRIFTS spectra of the samples following silylation, drying at 80 °C and calcination at 500 °C, in comparison to unmodified Na-ZSM-5. On treatment with BSTFA and drying, the broad band at 3615 cm^{-1} attributed to a combination of bridging hydroxyl groups and extra framework alumina species[28,29] apparently very slightly decreased in intensity with increasing silylation. Both the amide functionality at 3470, 3385 (-NH stretch, Figure

S4.1a) and 1745 cm^{-1} (C=O stretch, Figure S4.1b) appeared on the spectra with increasing intensity as silylation was increased, confirming functionalization of zeolite surface sites with BSTFA.

Silylated Na-ZSM-5 samples were then calcined at 500 °C to remove organic precursors and complete the surface modification process. High temperature treatment was required due to the known stability of silane groups. Thermal decomposition of organic species was noted by the complete removal of all amide region bands observed during prior functionalization with BSTFA (-NH stretch, Figure S4.1c and C=O stretch, Figure S4.1d). The more pronounced difference in the amide (-NH stretch) region of the SZ-2 spectra before and after calcination compared to SZ-0.6 (Figure S4.1e) is consistent with the greater concentration of silylating agent present. Notably, the silanol band at 3615 cm^{-1} was relatively unaffected by thermal treatment, indicating a permanent change in the surface exchange sites. This was thought to result from formation of new SiO_x species on the zeolite surface which are incapable of facilitating ion exchange, as observed by Ichikawa et al.[28]

Copper ion exchange was then performed on modified Na-ZSM-5 samples, followed by treatment in air at 500 °C to remove organic species. Figure S4.2 shows DRIFTS analysis of a selection of the modified Cu-ZSM-5 series. The silanol band at 3615 cm^{-1} was reduced in intensity following treatment with copper solution and calcination (Figure S4.2a). Reduction in 3615 cm^{-1} band intensity compared to modified Na-ZSM-5 precursors was noted even after calcination (Figure S4.2b), indicating a permanent change in the zeolite surface structure likely due to the inclusion of copper ions. Copper exchange was also accompanied by a color change in the catalyst from white to pale blue, with an identical appearance to normal unmodified Cu-ZSM-5.

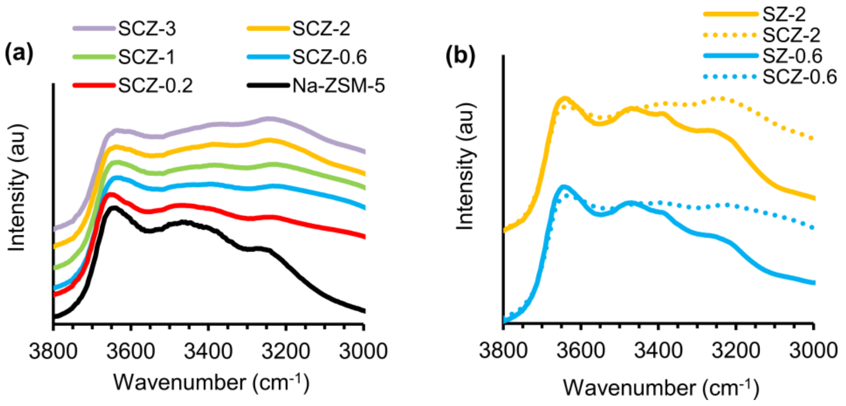

Figure S4.2 - DRIFTS spectra of BSTFA-modified Cu-ZSM-5 and unmodified Na-ZSM-5: (a) modified catalyst series after copper exchange and calcination at 500 °C; (b) comparison of selected modified Na-ZSM-5 samples (solid line) and corresponding modified Cu-ZSM-5 samples after copper exchange and calcination at 500 °C (dotted line).

S5 - Monitoring Cu-ZSM-5 Activation by UV-vis Spectroscopy

UV-vis spectroscopy was performed during the partial methane oxidation process to monitor the presence of the mono(μ-oxo) dicopper core, indicative of catalyst activity. Figure S5.1 shows the in situ UV-vis rig used. The UV-vis rig consists of a plug flow reactor mounted inside a tube furnace. A Hellma 668.006-UVS fibre optic probe was fixed outside the furnace and attached to a PerkinElmer Lambda 650S UV-vis spectrometer. Flexible tubing allowing placement of the reactor within the furnace for activity tests, or at a distance of 1 mm under the probe for UV-vis analysis. A dark box was used around the probe to prevent saturation of the detector with natural light. Mass flow controllers were used to regulate the flow of all gases. Gas streams were dried with in-line silica moisture traps and a central cold trap containing CO_2/acetone at -79 °C. Temperature was controlled by a thermocouple mounted inside the catalyst bed.

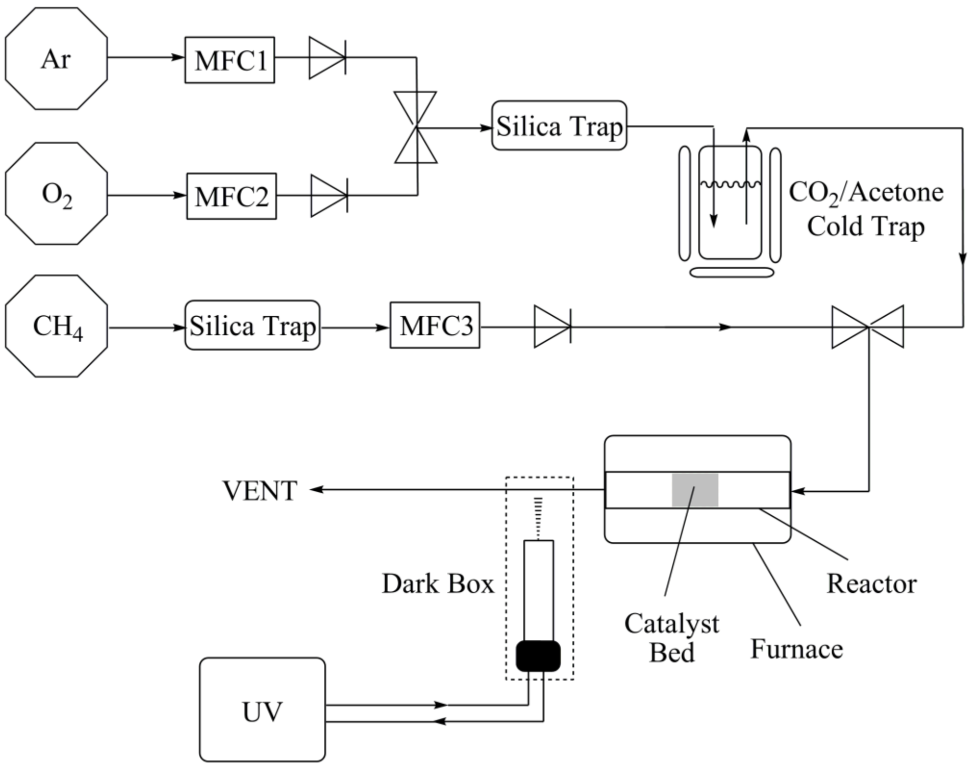

Figure S5.1 - Fibre optic probe, UV-vis spectrometer and quartz plug-flow reactor rig

The following experimental method was used as standard. Pre-calcined, pelletized Cu-ZSM-5 samples (0.7 g, 250-500 µm pellets) were loaded into the quartz reactor, secured with quartz wool, and aligned in the *in-situ* UV-vis rig. Samples were initially pre-reduced at 500 °C for 3 hours under Ar. The pre-reduced samples were then activated overnight in 100% O_2 (50 ml/min, 500 °C). Samples were cooled in an oxygen atmosphere and flushed with Ar (20 ml/min, 5 mins) to prevent the formation of explosive mixtures. Subsequently, CH_4 (50 ml/min, 1% in Ar) was passed over the activated samples for 1 hour while heating to 150 °C (10 °C/min), then the system was cooled and flushed again with Ar at ambient temperature.

UV-vis spectra were recorded throughout the above method:

- Initially before catalyst activation
- At ambient temperature after activation in O_2
- At ambient temperature after reaction with CH_4

Spectra were recorded from 600 to 300 nm in reflectance (% R) mode using the parent zeolite NH_4-ZSM-5 as background. Normalisation was performed to the minimum absorption point of each spectrum (typically 550 nm). Raw spectra were individually converted and presented as absorption spectra (A) using the Kubelka-Munk transform. Final absorption spectra as presented were the result of the superposition of 8 scans. A typical set of spectra for CZ-2.65 is shown in Figure S5.2.

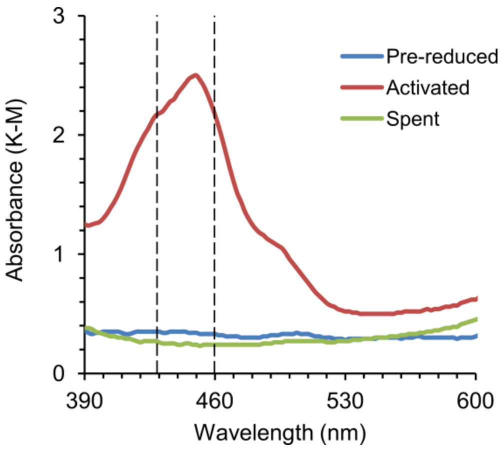

Figure S5.2 - UV-vis spectra of CZ-2.65 following pre-reduction in Ar, activation in O_2, then reaction with CH_4. The absorption peak between 430-460 nm is indicated.

S6 - Probe Molecule Adsorption Study by Transmission FTIR

Transmission FTIR was used in conjunction with the adsorption of probe molecules to monitor the distribution of copper on samples of Cu-ZSM-5. Figure S6.1 shows the IR gas adsorption rig used. The IR rig consists of a heatable Specac gas exchange sample holder (GESH) fitted with ZnSe windows and mounted on a Bruker Tensor 27 IR spectrometer. Mass flow controllers were used to regulate the flow of all gases, allowing adsorption of gaseous and vapor probe molecules (via bubbler) to the sample. Gas streams were dried with in-line silica moisture traps and heated separately to prevent vapor condensation. An isolable vacuum pump allowed outgassing or purging of the cell during or between experiments. Temperature was controlled by a thermocouple mounted on the cell exterior.

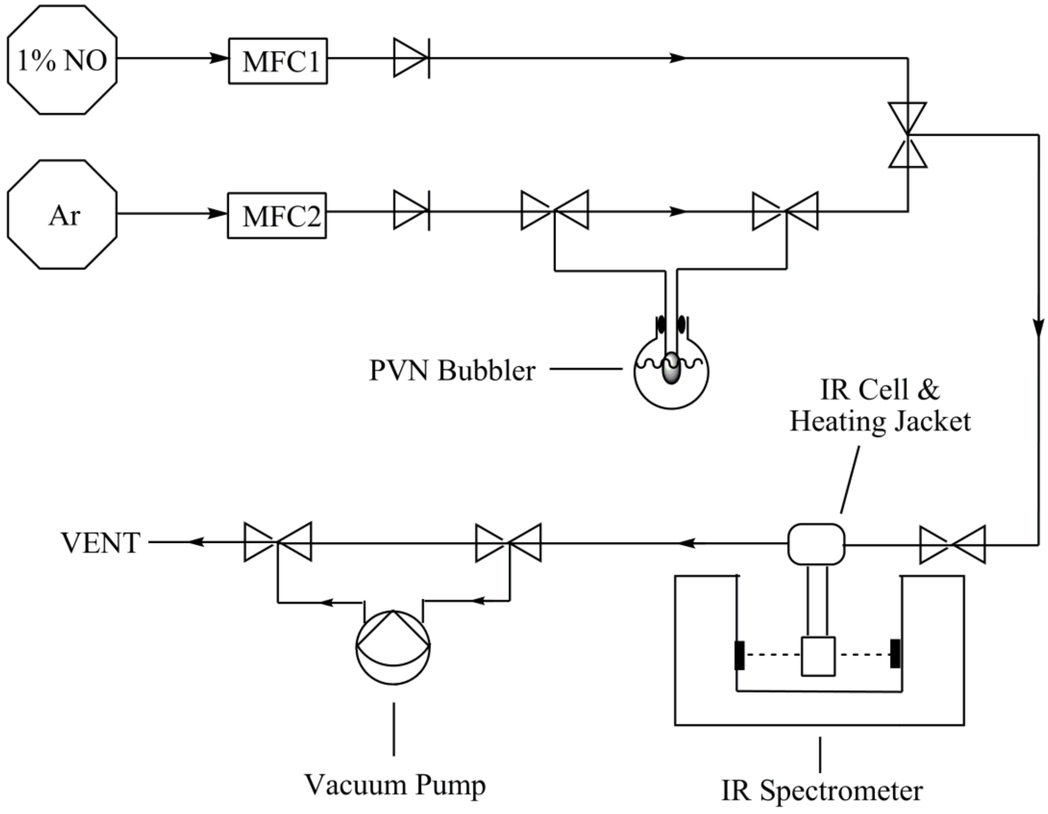

Figure S6.1 - In situ transmission IR cell and spectrometer rig assembly

The following experimental method was used as standard. Cu-ZSM-5 samples (20 mg) were pressed into self-supporting discs at 5000 kg for 1 minute using a 13 mm stainless steel die. The discs were sealed in a gas adsorption cell and aligned within the spectrometer. Samples were initially outgassed under vacuum at 150 °C for 2 hours. Pivalonitrile (PVN) vapour in a bubbler at 25 °C was then adsorbed to the sample via Ar stream (25 ml/min) at 40 °C for 30 mins, before flushing the cell with pure Ar until a stable IR signal for PVN was observed. NO

(25 ml/min, 1% in Ar) was subsequently adsorbed at 40 °C overnight, before again flushing with Ar to remove excess gaseous NO until a stable signal was observed. Blank experiments were performed by adsorption of PVN then NO to the parent zeolite Na-ZSM-5, these were used to identify features resulting from the presence of copper on the samples.

IR spectra were recorded throughout the gas exchange process:

- Initially before probe molecule adsorption
- During PVN adsorption and Ar flushing
- During NO adsorption and flushing

Before analysis of each catalyst sample, a dry KBr disc (20 mg) was loaded into the cell and scanned to provide the baseline. Spectra were obtained from 400 to 4000 cm^{-1} with a resolution of 4 cm^{-1}. Each spectra was the result of 128 scans. Data processing was performed using the OPUS software package. All spectra were manually baseline corrected and normalized to the zeolite overtones at 1950-2050 cm^{-1}, except following NO adsorption, where normalization was performed at the higher energy band (centred at 2000 cm^{-1}). Where multiple overlapping peaks were observed in the PVN and NO absorption range, spectra were deconvoluted by peak fitting using Gaussian curves. Data was therefore presented as a result of integration of the peak fitted curves. The area of the curves was directly related to the intensity of the absorption bands observed and therefore to the concentration of absorbing species, according to the Beer-Lambert law.

An example of the formation of relevant IR bands during PVN absorption is outlined in Figure S6.2. An example of the deconvolution and peak fitting process for PVN-Cu^{2+} and NO-Cu^{2+} is shown in Figure S6.3 and S6.4 respectively.

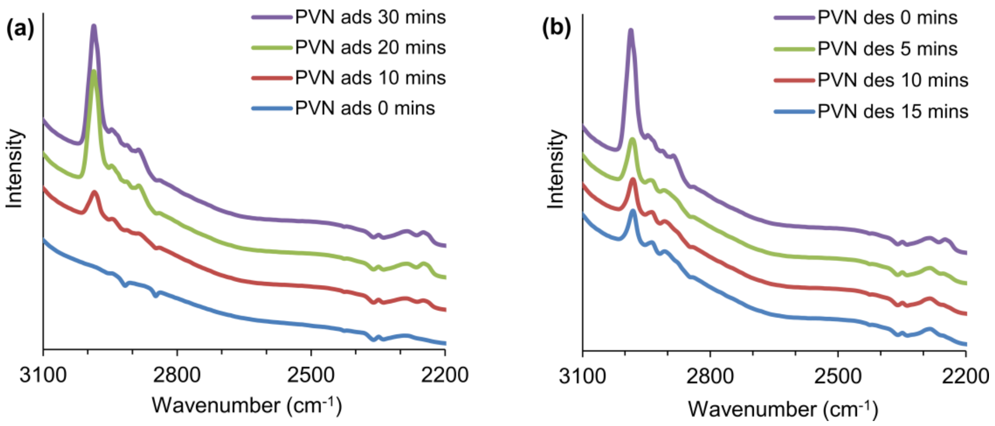

Figure S6.2 - Transmission IR spectra of CZ-1.52 showing alkane (-CH) and nitrile (-CN) stretching regions: (a) during PVN adsorption, (b) while flushing Ar following PVN adsorption.

In Figure S6.3, the various component absorption bands were identified[19] as follows:

2298 cm^{-1} - interaction of PVN with zeolite Al^{3+} sites (strong)
2282 cm^{-1} - interaction of PVN with Cu^{2+} (strong)
2266 cm^{-1} - interaction of PVN with surface silanol groups (weak)
2244 cm^{-1} - interaction of PVN with surface silanol groups (weak)

During analysis of Na-ZSM-5 no band at around 2280 cm^{-1} was observed, this band was thus attributed to copper. The remaining very intense bands at 2248 and 2238 cm^{-1} were attributed to zeolite silanol and Al^{3+} interactions. The shift may be due to the absence of copper on zeolite exchange sites, which are integrally linked to Al^{3+} in the zeolite structure.

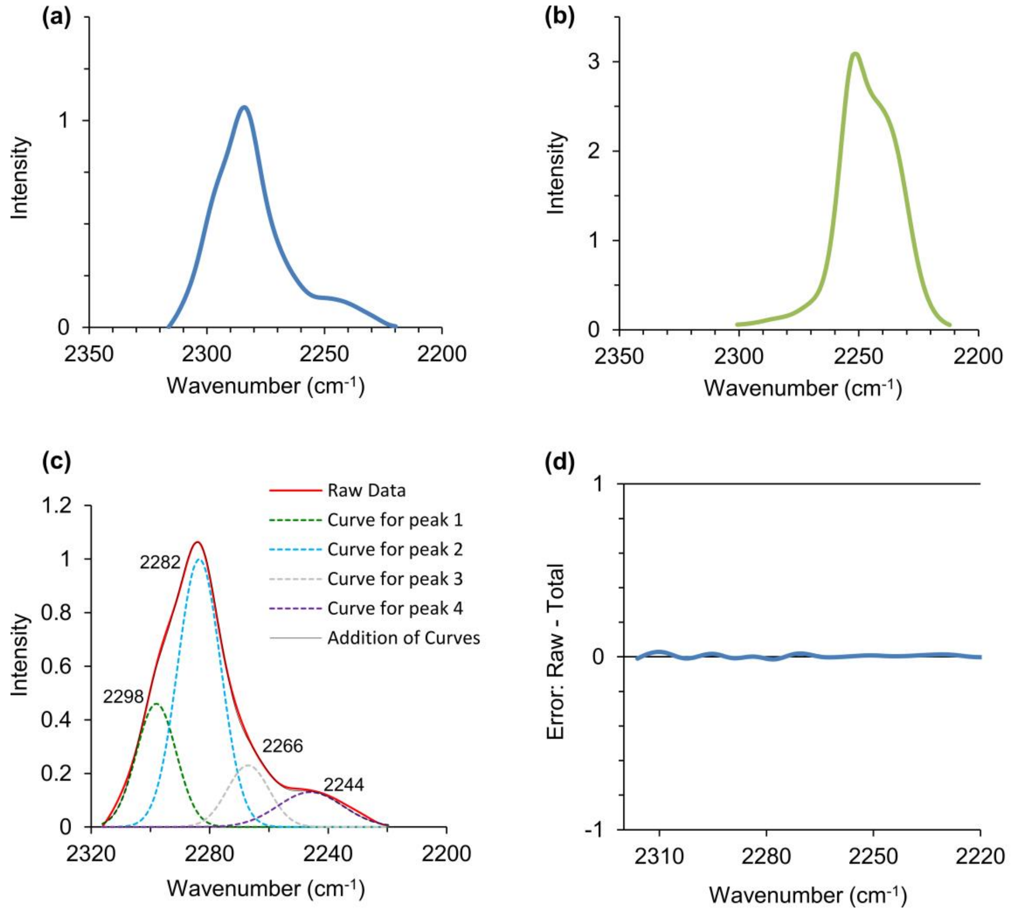

Figure S6.3 - Normalised, baseline-corrected transmission IR spectra showing detailed -CN stretching range after PVN adsorption and Ar flush on: (a) CZ-2.12. (b) Na-ZSM-5. (c) Deconvolution of PVN absorption bands from CZ-2.12 using Gaussian function. (d) Peak matching of raw data to final deconvoluted peaks, error shown on y-axis.

In Figure S6.4, the various component absorption bands were identified[19] as follows:

1918 cm^{-1} - unidentified (weak)
1907 cm^{-1} - interaction of NO with Cu^{2+} (strong)
1892 cm^{-1} - interaction of NO with surface silanol groups (strong)
1880 cm^{-1} - unidentified (weak)

During analysis of Na-ZSM-5, bands were observed at approximately all the same wavenumbers except for 1907 cm^{-1}, which was therefore assigned to NO-Cu^{2+} interactions.

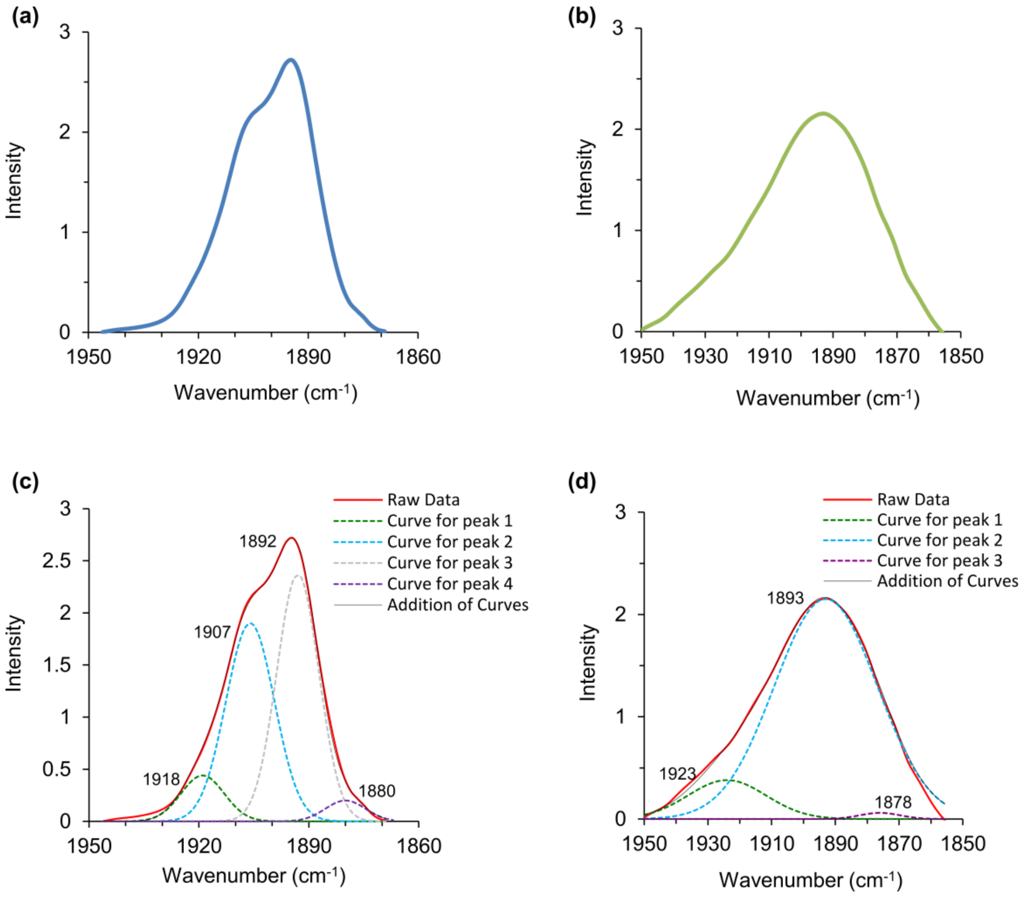

Figure S6.4 - Normalised, baseline-corrected, background-subtracted transmission IR spectra showing detailed NO stretching range after PVN adsorption and Ar flush on: (a) CZ-2.12. (b) Na-ZSM-5. Deconvolution of NO absorption bands from (c) CZ-2.12 and (d) Na-ZSM-5 using Gaussian function.

S7 - Activity Testing of Standard and Modified Cu-ZSM-5 for Partial Methane Oxidation

Activity testing during the activation and reaction phases was carried out in the UV-vis rig detailed earlier Figure S5.1. The following method was used as standard. Pre-calcined, pelletized Cu-ZSM-5 samples (0.7 g, 250-500 µm pellets) were placed in a quartz plug flow reactor, secured with quartz wool and aligned in the UV-vis rig. Activation was performed overnight in oxygen (50 ml/min, 500 °C). Samples were cooled to room temperature, flushed with argon and then exposed to methane (50 ml/min, 1% in Ar) at 150 °C for 1 hour (10 °C/min). The system was cooled and flushed again with argon at room temperature. The products remained adsorbed to the catalyst surface. Two different analytical methods were used on spent samples following activation and reaction with methane: (i) reaction products were directly extracted in aqueous solution and analyzed by GC, (ii) temperature programmed oxidation (TPO) was performed and combustion products monitored by MS.

(i) Product Analysis - GC Extraction Method

Samples were removed from the reactor and the products extracted by stirring vigorously in deionized H_2O (1.5 ml) for 24 hours. The resulting suspension was centrifuged using an Eppendorf 5702 (5 mins, 2000 rpm), the aqueous solution passed through a syringe filter (13 mm, 0.45 µm nylon membrane) and analyzed by GC. Analysis was conducted with a PerkinElmer Clarus 500 GC equipped with an FID (250 °C) and a Supelco 25353 Carbowax® Amine column (30 m x 530 µm, 1 µm film). 2 µl samples were injected at 150 °C, while the column was heated from 60-120 °C (10 °C/min). Products were identified by matching retention times with spiked samples of known compounds. Product concentrations were calculated by the calibration curve method. Due to the highly dilute nature of the products, prior to analysis of catalyst washings the injector and column were cleaned thoroughly with 8 individual samples of deionized H_2O to provide a clear baseline measurement.

(ii) Product Analysis - TPO Method

TPO was performed in the MS rig detailed in Figure S7.1. The MS rig consists of a quartz plug flow reactor mounted in a tube furnace, with a Hiden Analytical mass spectrometer located downstream for in situ analysis of products. Mass flow controllers were used to regulate the flow of all gases. A remote operated 4-way valve connected to a gas switchboard allowed pre-mixing of desired flow compositions before contact with the sample. Temperature was controlled by a thermocouple mounted inside the catalyst bed.

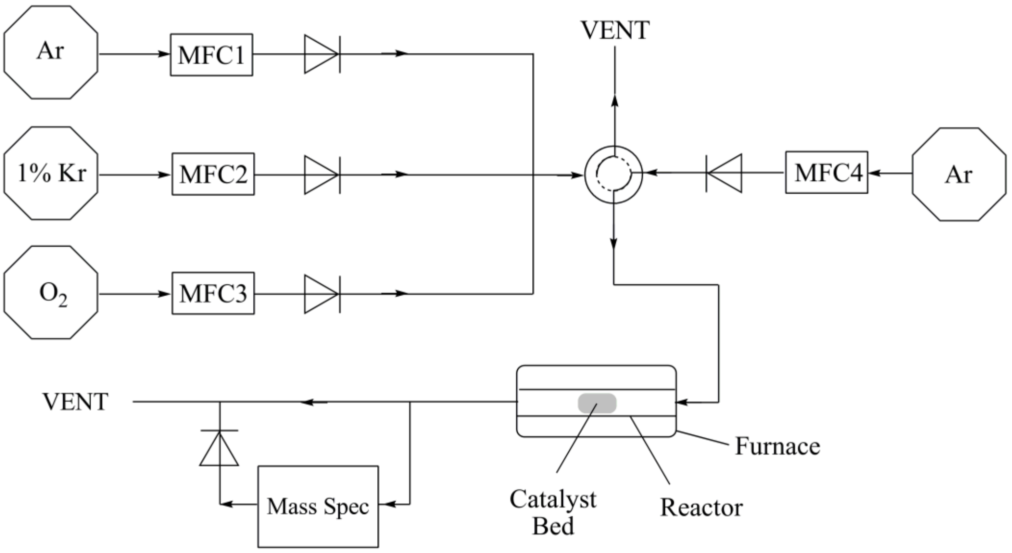

Figure S7.1 - MS rig for in situ TPO studies

Before analysis, the plug-flow reactor was removed from the UV-vis rig and transferred to the MS rig in an inert environment. A feed of O_2 (100 %) was initially introduced with Kr (5% in Ar) as internal standard (Kr/O_2 10/40 ml/min) and passed at ambient temperature for 30 mins to confirm a stable MS signal. The catalyst was then heated from 20 to 500 °C (10 °C/min) and the appearance of combustion products monitored by MS, the maximum temperature was maintained for 15 mins to confirm the complete desorption of any products. For the unmodified series, blank TPO experiments were performed as above but with activation in Ar (rather than O_2), followed by reaction with methane. The CO_2 detected as a result of adsorbed methane was subtracted from that of 'active' TPO experiments to give the final CO_2 yield.

Raw mass spectra were baseline corrected and normalized using the Kr trace as an internal standard. Normalised traces were smoothed to an average of ten data points and the relevant product desorption curves integrated to quantify the raw product. The data was converted to molar values using the response factor for each product as determined by calibration curve. Final data was therefore presented as molar product yield per gram of catalyst. MS was performed using the following system parameters: Total flow rate = 50 ml/min. Internal standard = Kr, 1% of total flow. Electron emission current = 20 mA. Electron energy = 70 V. Input Mode = SEM at 1950 V. Traces (m/z): CH_4 (15), O_2 (16), H_2O (18), CO/N_2 (28), CH_3O^+ (31), Ar (36), CO_2/N_2O (44), DME (45), Kr (82). Relative SEM = 1, except CH_3O^+ (31) relative SEM = 0.1.

S8 - Additional Data

(i) - Methanol Yield as a Function of Specific Copper Distribution

Figure S8.1 shows CH_3OH yield from aqueous extraction as a function of internal and external copper loading, characterised by FTIR and adsorption of probe molecules. Analysis of product yield from aqueous extraction (µmolg^{-1} zeolite) in relation to specific copper distribution (NO-Cu^{2+}, PVN-Cu^{2+} IR bands) confirmed that external based copper sites characterized by PVN adsorption have no correlation to methanol yield. This was previously observed for unmodified catalysts[19] but extends to this study of surface modification. In contrast, intraporous copper sites characterized by NO adsorption have a clearly defined relationship to product yield for the unmodified series (Figure S8.1c), however no trend was observed for the modified series (Figure S8.1d).

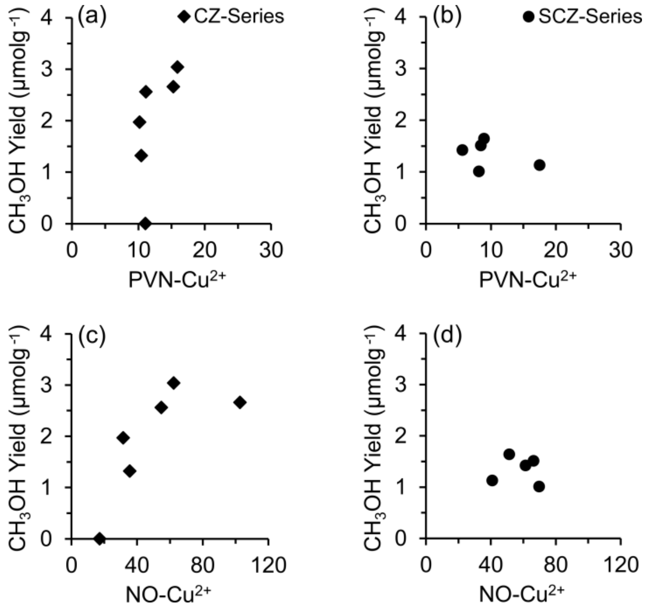

Figure S8.1 - Methanol yield from aqueous extraction as a function of specific copper distribution on Cu-ZSM-5, characterized by integrated IR peaks following PVN and NO adsorption. Yield shown for CZ series in relation to: (a) PVN-Cu^{2+}; (c) NO-Cu^{2+}; and for modified SCZ series in relation to: (b) PVN-Cu^{2+}; (d) NO-Cu^{2+}.

(ii) - CO_2 Yield as a Function of Specific Copper Distribution

Figure S8.2 shows CO_2 yield from TPO of unmodified catalysts as a function of the internal and external copper loading, characterised by NO and PVN adsorption respectively. It is suggested that external copper has no influence on the yield of CO_2 observed, indicating that it is not active for methanol production. On the other hand, the amount of internal copper corresponds to a general increase in the volume of CO_2 obtained.

Compared to the yield observed from aqueous product extraction, the results confirm that at higher Cu loadings a significant portion of the reaction products remain trapped in the zeolite pores and are not recoverable by normal aqueous extraction. Furthermore, similarly to that reported by Bitter et al.[19] the amount of product evolved directly correlates to intraporous copper sites as characterized by NO adsorption (Figure S8.2b), while no correlation was noted in relation to external copper sites probed by PVN adsorption (Figure S8.2a). As expected, only copper sites in the zeolite channels were found to be potentially active for partial methane oxidation. For the modified catalysts, the CO_2 yield was proportional to the amount of intraporous copper species (Figure S8.2d), but showed an inverse relationship to the amount of external sites (Figure S8.2c).

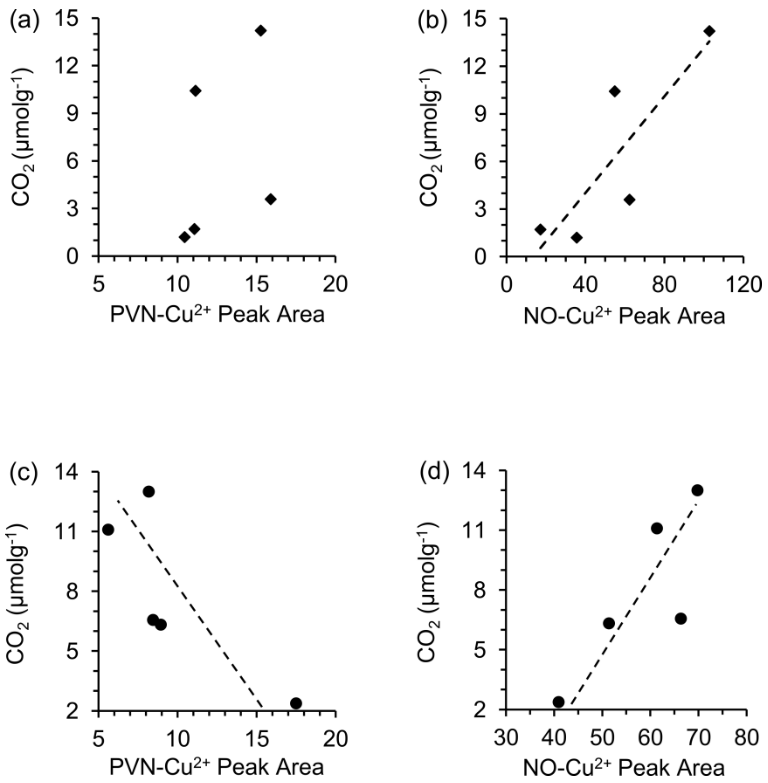

Figure S8.2 - CO_2 evolved during TPO of Cu-ZSM-5 samples at 500 °C in O_2. CO_2 yield shown as a function of integrated IR absorption bands observed following adsorption of PVN (representative of external copper sites) to: (a) CZ-series and (c) SCZ-series; and adsorption of NO (representative of intraporous copper sites) to: (b) CZ-series and (d) SCZ-series.

(iii) - Methanol / CO_2 Yield as a Function of Silylation

For the modified SCZ series, the influence of silylation level on the amount of products observed by both aqueous extraction and TPO is shown in Figure S8.3. Although the silylation process resulted in an overall decrease in the amount of aqueous desorbed methanol compared to the unmodified catalysts, this was not dependent on the level of silylation (Figure S8.3a). Conversely, a clear influence was observed on the amount of CO_2 evolved (Figure S8.3b). At an ideal level of between 1 to 2 wt.% SiO_2 present on the zeolite surface, the CO_2 yield reached a maximum, indicating a large amount of adsorbed methanol present within the zeolite pores. The large increase in CO_2 yield from 1 to 2 wt.% SiO_2 is consistent with structural characterization, which showed a greatly increased NO:PVN ratio during gas adsorption studies. However product desorption in the aqueous phase remained significantly hindered, similarly to the unmodified catalysts tested. In combination with TON data (see main paper), this suggests a more active catalyst as a result of silylation.

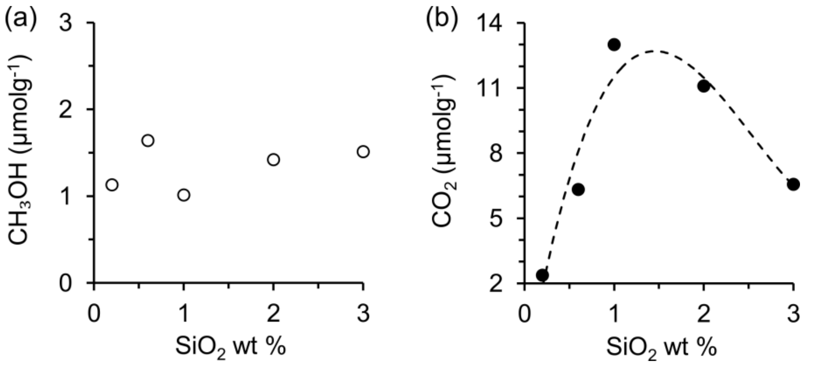

Figure S8.3 - Activity data for modified SCZ-series catalysts: (a) CH_3OH yield after aqueous product extraction as a function of total silylation (SiO_2 wt.%); (b) CO_2 evolved during TPO at 500 °C in O_2 as a function of total silylation.

(iv) - CO_2 Yield as a Function of UV-vis Intensity

Figure S8.4a shows the difference in aqueous extracted methanol and TPO desorbed CO_2 from the modified catalyst series as a function of total copper loading. It is clear that CO_2 released is in excess of any aqueous products, but there is no clear correlation with copper loading. This is in contrast to the amount of silylation (Figure S8.3). Figure S8.4b shows that unlike the unmodified catalysts where a correlation was observed between the CO_2 evolved during TPO and the UV-vis signal, in the case of the modified catalysts no clear correlation was found between the UV-vis signal intensity and the CO_2 evolved during TPO analysis. This implies that for these silylated catalysts, similarly to the MOR, BEA and FAU catalysts,[11] the intensity of the UV-vis signal does not directly represent the amount of active core sites when compared to unmodified ZSM-5. Therefore monitoring of CO_2 evolution by TPO and the volume of extra- and intraporous copper sites by IR were considered as potentially more accurate indicators of catalytic activity.

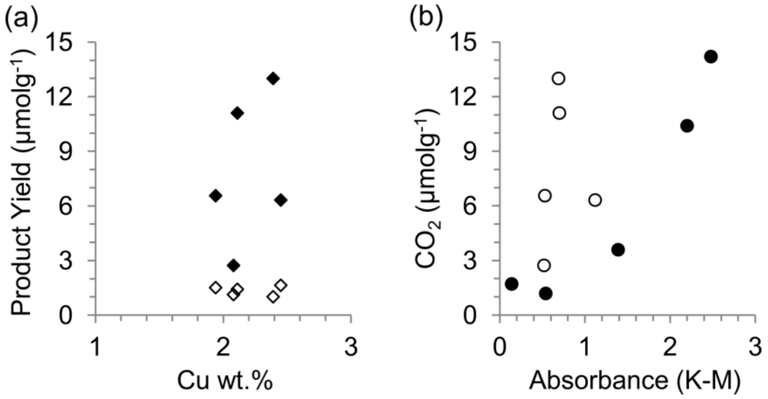

Figure S8.4 - (a) Data for modified SCZ-series catalysts, CH_3OH yield from aqueous extraction (hollow points) and CO_2 evolved during TPO at 500 °C in O_2 (solid points) as a function of total copper loading; (b) CO_2 evolved as a function of UV-vis intensity for unmodified (solid points) and modified (hollow points) series.

(v) - Effect of Exchange Time on Copper Loading

Total copper loading observed with respect to the contact time of copper(II) acetate and Na-ZSM-5 during wet ion exchange is shown in Figure S8.5. The data presented is for CZ-2.65, indicating that the majority of copper detected was present after just 1 hour. This suggests rapid saturation of external and pore-mouth exchange sites, with slower diffusion deep into the zeolite pores accounting for the increased loading over time.

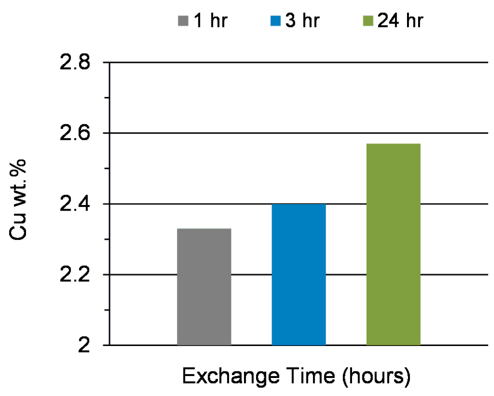

Figure S8.5 - Illustration of effects on total copper loading (Cu wt.%) with increasing exchange time during preparation of CZ-2.65. Experimental values determined through ICP.

(vi) - Gaussian Calculations of BSTFA

Gaussian calculations of a single BSTFA molecule were performed to determine the approximate molecular diameter in relation to the pores of ZSM-5. As shown in Figure S8.6, the results confirmed that BSTFA (9.304 Å) was too large to enter the micropore structure ([100] 5.1 x 5.5, [010] 5.3 x 5.6 Å) and was therefore expected to perform surface modification selectively on the external zeolite exchange sites. Calculations were performed using Gaussian '09 software package with B3LYP functional and 6-311G+dp basis set.

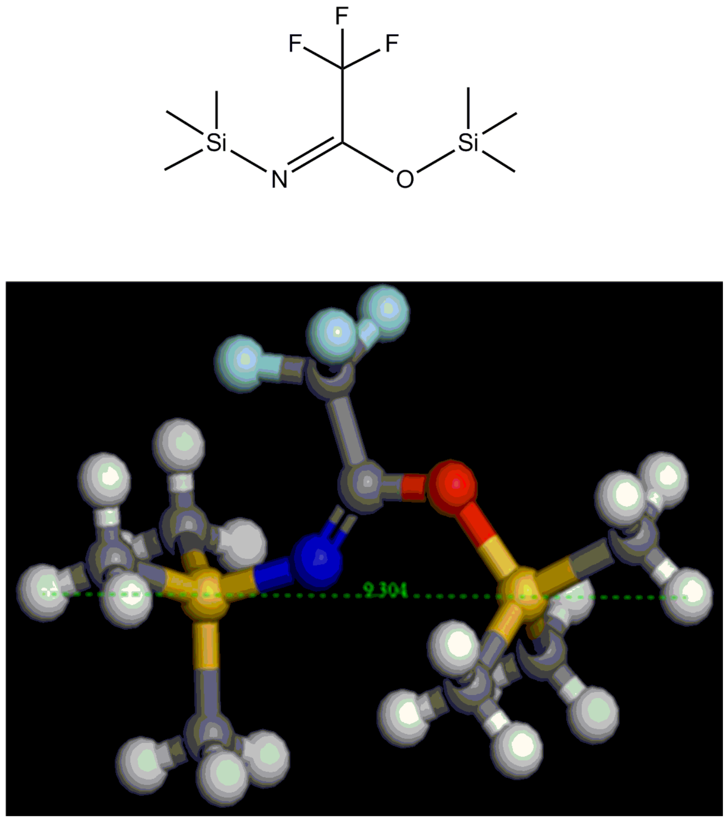

Figure S8.6 - Illustration of BSTFA molecule and corresponding Gaussian calculation.
